# Global cropland could be almost halved: Assessment of land saving potentials under different strategies and implications for agricultural markets

**DOI:** 10.1371/journal.pone.0263063

**Published:** 2022-02-22

**Authors:** Julia M. Schneider, Florian Zabel, Franziska Schünemann, Ruth Delzeit, Wolfram Mauser

**Affiliations:** 1 Department of Geography, Ludwig-Maximilians-Universität München, Munich, Germany; 2 Department of Bioeconomy and Computational Science Lab, University of Hohenheim, Stuttgart, Germany; 3 Department of Environmental Sciences, University of Basel, Basel, Switzerland; 4 Kiel Institute for the World Economy, Kiel, Germany; University of Naples Federico II: Universita degli Studi di Napoli Federico II, ITALY

## Abstract

The pressure on land resources continuously increases not only with the rising demand for agricultural commodities, but also with the growing need for action on global challenges, such as biodiversity loss or climate change, where land plays a crucial role. Land saving as a strategy, where agricultural productivity is increased to allow a reduction of required cropland while sustaining production volumes and meeting demand, could address this trade-off. With our interdisciplinary model-based study, we globally assess regional potentials of land saving and analyze resulting effects on agricultural production, prices and trade. Thereby, different land saving strategies are investigated that (1) minimize required cropland (2) minimize spatial marginalization induced by land saving and (3) maximize the attainable profit. We find that current cropland requirements could be reduced between 37% and 48%, depending on the applied land saving strategy. The generally more efficient use of land would cause crop prices to fall in all regions, but also trigger an increase in global agricultural production of 2.8%. While largest land saving potentials occur in regions with high yield gaps, the impacts on prices and production are strongest in highly populated regions with already high pressure on land. Global crop prices and trade affect regional impacts of land saving on agricultural markets and can displace effects to spatially distant regions. Our results point out the importance of investigating the potentials and effects of land saving in the context of global markets within an integrative, global framework. The resulting land saving potentials can moreover reframe debates on global potentials for afforestation and carbon sequestration, as well as on how to reconcile agricultural production and biodiversity conservation and thus contribute to approaching central goals of the 21st century, addressed for example in the Sustainable Development Goals, the Paris Agreement or the post-2020 global biodiversity framework.

## Introduction

With rising global demand for agricultural commodities for food, feed, bioenergy, and the emerging bioeconomy, the pressure on land as a resource and production factor continuously increases [1–4]. At the same time, land for biodiversity conservation, carbon sequestration and further ecosystem services is crucial to tackle main challenges of the 21^st^ century, such as climate change and biodiversity loss, which are also addressed in the Sustainable Development Goals [5]. Land use competition and inherent trade-offs are thus becoming an increasingly important research subject [6–9].

Improving the efficiency of land use in agricultural production systems by increasing the crop production per unit of cultivated land is one strategy to address this land use trade-off. It is often referred to as the Borlaug hypothesis, according to which achieving higher yields results in agricultural land being saved and thus freed up for other uses [10, 11]. In particular, the resulting potential for biodiversity conservation is controversially discussed within the debate on land-sparing vs. land-sharing [12–17], as there is also clear evidence of negative effects of agricultural intensification on biodiversity and ecosystems, such as freshwater depletion, soil erosion, increasing greenhouse gas emissions, habitat homogenization or the loss of habitat availability for wild species [18–27].

Indeed, research findings indicate high potentials for agricultural intensification and optimization of crop and farm management [28–32]: Global crop production on currently cultivated land could be more than doubled [28] and a recent study from Folberth et al. [33] suggests that closing yield gaps would enable to take nearly 50% of current global cropland out of production.

However, land use and cropping patterns are strongly intertwined with economics and policy: Within an ongoing commercialization and globalization of agricultural systems, spatially distant drivers and interconnections gain importance and global crop prices and trade flows affect local land use decisions and cropping patterns [8, 34–36]. Additionally, land use patterns are embedded within their regional socio-economic framework, and shaped by regional drivers such as land use regulations and policies [13, 37], population and consumption changes [38], and can play a crucial role regarding regional societal structures and livelihoods [39]. When investigating the potential of agricultural intensification to save cropland, it is thus important to consider the interaction and feedback between local land use decisions and global markets, as well as to account for regional markets and demand structures.

Previous research on land saving is mainly based on statistical data: Studies either focus on past potentials, based on the evaluation of the correlation between yield increase and cultivated area over time [37, 40, 41], or investigate future potentials via extrapolation of statistics, that thus strongly depend on assumptions on future development paths of demand, dietary patterns, and yield increase [42]. Model-based assessments of land saving potentials are rare [33, 43, 44], and even though economic and societal implications and feedbacks on land saving are widely discussed [8, 36], their quantitative effect so far remains understudied.

Our model-based study aims to evaluate the potential of land saving and analyze the resulting effects on agricultural markets from an interdisciplinary perspective by coupling a process-based biophysical crop model and a computable general-equilibrium (CGE) model of the world economy. In a first step, we globally assess the potential of agricultural intensification to reduce the required cropland for current agricultural production on a regional scale by referring to the simulated yield potentials of the crop model. The resulting land saving and yield potentials are then integrated into the CGE model to investigate in a second step the impact of land saving on agricultural markets in terms of changing crop prices, production volumes and trade flows on a regional and global scale.

The land saving potential is assessed under three land saving strategies that differ in their main driving factor: (1) A production-optimized strategy that is solely driven by biophysical yield potentials to minimize required cropland area (2) a policy-driven land saving strategy that aims to counteract a spatial concentration of land saving to avoid creating or increasing spatial inequalities, and (3) a profit-optimized implementation of land saving that considers biophysical and socio-economic aspects to maximize the value of production. These three different strategies lead to different spatial patterns of land saving, which allows us to investigate, how different assumptions on the drivers and the spatial implementation of land saving affect its potential.

By applying our integrative coupling approach, we can quantitatively link the three land saving scenarios with their global and regional economic implications which are derived from the CGE model, such as changes in staple crop prices or shifting import and export patterns of specific crops. This makes it possible to investigate the socio-economic effects of land saving, for example on food security, in a more comprehensive and differentiated way, which is crucial to evaluate potentials and challenges of land saving to contribute to a more efficient and sustainable use of land as a resource.

## Materials and methods

We refer to the term ‘land saving’ [8, 11, 40] to describe the potential reduction of required cropland to reach a defined reference production under the assumption of agricultural intensification. Contrary to the term ‘land sparing’, where freed up land is used for biodiversity conservation, ‘land saving’ does not predefine the usage of land that could potentially be taken out of agricultural production [11].

### Conceptual framework

Within this study, the land saving potential is assessed for 15 globally important agricultural food and energy crops that together represent 70% of global cropland area and 65% of global crop production [45] (S2 Appendix). Our model framework is spatially structured into 17 regions, that are divided into sub-regions according to Agro-Ecological Zones (AEZs) [46, 47] (S3 Appendix). The sub-regions account for the heterogeneity of the primary production factor land by reflecting the varying productivity characteristics for agriculture within a region regarding environmental and climatic conditions [48]. Land saving and impacts on agricultural markets are analyzed at sub-regional level. This spatial structure allows, firstly, to capture differences in (sub-)regional production factors. Secondly, by maintaining sub-regional production volumes as reference production, we account for the relevance of regional agricultural production and specific crops for local economies, employment, culture and society. Thirdly, we can thereby account for linkages across different spatial scales [49, 50], considering local and regional conditions as well as global-scale dynamics and distant drivers.

### Models and data

We refer to statistical data on harvested areas and yields from the Global Trade Analysis Project (GTAP) 9 database [51] to determine current crop production as reference production target to be achieved in each sub-region. Holding crop-specific production volumes constant at current levels allows us to focus on land saving and differences between the strategies by avoiding an overlapping with effects resulting from assumptions on the future development of agricultural production.

Potential yields under local climate and environmental conditions are derived from Mauser et al. [28]. Based on simulations with the biophysical process-based crop model PROMET (S1 Appendix), they describe biophysical yield potentials attainable under 1981 to 2010 climate conditions. Therefore, perfect crop management conditions, e.g. regarding sowing and harvest, fertilization, pest and disease control, and a realization of multiple harvest potentials are assumed. The potential yields are provided for rainfed and irrigated conditions separately as 30-year mean at representative sample locations within each sub-region for the 15 considered agricultural crops (S4 Appendix). To be consistent with the coarser representation of crops in the economic model, the biophysical yield potentials are grouped into 9 crop categories (Table in S2 Appendix). Furthermore, we assume that yield gaps are closed by 80%, to take factors into consideration that limit the realization of potential yields (S4 Appendix).

To account for the interplay of agricultural markets and land saving, we apply the CGE-model DART-BIO that represents the world economy in our integrative approach. DART-BIO is a multi-sectoral model that includes all production and consumption linkages through product and factor markets in the global economy and simulates the interplay of demand and supply through a system of nonlinear equations based on Walrasian general equilibrium theory. The model is calibrated to the GTAP 9 database [51] and includes 52 sectors with 10 dedicated crop production sectors that use intermediate inputs and production factors in the form of labor, capital and 18 land types corresponding to the above-mentioned AEZs. An implementation of land saving changes two economic parameters in the model: The land productivity increases and the land endowment decreases. In DART-BIO these two effects are implemented by a productivity shock on the land input in the production function of agricultural crops on the one hand, and by decreasing the availability of the production factor land for each crop according to the results of the land saving assessment on the other hand. This results in two opposing effects on input costs: While increased productivity makes the land input relatively cheaper, the reduction of cropland makes the land input relatively more expensive compared to other inputs (as land becomes scarcer). For most crops and regions, the productivity shock dominates in the net effect, so that producers have lower average input costs for land. While land prices decline, the costs of other inputs, such as labor, capital, and intermediates such as fertilizer, increase, partly representing intensification costs. Under the perfect competition assumption in our model, this leads to decreasing output prices and thus also lower consumer prices of crops. Consumers react to lower prices with higher demand, which motivates producers to increase their output. An increase in production means an increase in producer demand and competition for scarce inputs such as land, labor and capital, which increases input prices and as such crop production prices. Consumer demand decreases as prices for crops rise. This interplay happens simultaneously on factor and product markets until prices are found where demand equals supply on all markets. With DART-BIO, we are thus able to model the through land saving induced supply shock, the resulting demand response and subsequent interplay between demand and supply on markets until a new market equilibrium is reached. In this approach, costs of intensification are only partly considered via higher input costs of other inputs than land. Additional costs, such as investment costs and costs for research and development, are not taken into account. Considering regional socio-economic conditions (e.g. consumption patterns, land regulations, economic policies) as exogenous factors and the relation between supply and demand, DART-BIO furthermore provides crop category-specific marginal profit functions for each sub-region. The functions describe the (marginal) profit that can be achieved by growing a certain crop on an additional unit of land, depending on the productivity of land as primary production factor in relation to other primary factor inputs such as labor and capital (S5 Appendix). This information is used to simulate the socio-economic land saving strategy (see below).

### Land saving strategies

**Fig 1:**
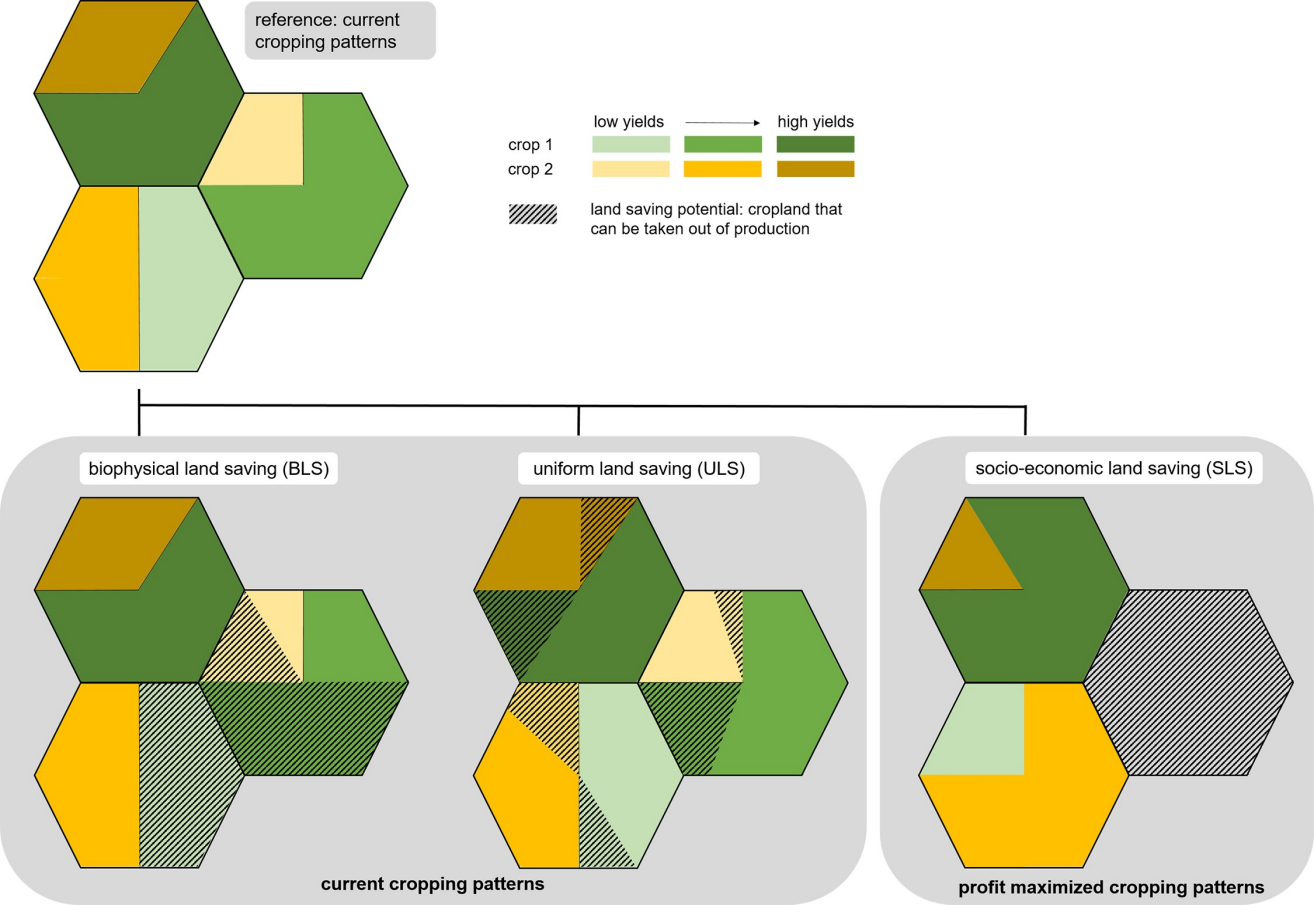
Schematic overview of the three different land saving strategies biophysical land saving (BLS), uniform land saving (ULS) and socio-economic land saving (SLS). The strategies differ in their assumptions on maintaining current cropping patterns (BLS, ULS) versus their change towards profit-optimized cropping patterns (SLS), and the spatial implementation of land saving at locations with the lowest yields (BLS), uniformly across the region at high- and low-yielding locations (ULS), or the least profitable locations (SLS).

We developed three algorithms to evaluate the potential of different implementation strategies of land saving (Fig 1). The algorithms differ in their main driving factor and in their initial assumption to maintain current cropping patterns, defined as the current crop-specific spatial location of cropland:

1. Biophysical land saving (BLS) that minimizes required cropland
2. Uniform land saving (ULS) that minimizes spatial marginalization
3. Socio-economic land saving (SLS) that maximizes the attainable profit

Within all three strategies, we preclude expansion of total cropland and of irrigated land by setting their maximum extent and spatial location to current cropland distribution and irrigation infrastructure according to Portmann et al. [52]. The algorithms operate at sub-regional scale to assess the land saving potential.

#### (1) Biophysical land saving

The BLS strategy assumes that the implementation of land saving is based solely on biophysical yield potentials under current cropping patterns. Therefore, the agricultural production of each crop category is concentrated on its current cropland with the highest potential yields, saving land at biophysically less productive current growing locations with low yield potentials (Fig 1). Thus, the production per area is optimized and the resulting land saving potential serves as an upper biophysical benchmark to minimize the required cropland for current production under current cropping patterns.

To simulate this strategy, we developed an allocation algorithm that, starting from locations with the highest potential yields within the sub-region for a crop category, successively accumulates the attainable production at each current cropland location under potential yields. By accumulating production along the potential yield gradient and stopping when the production target is reached, the lowest-yielding cropland for each crop category within a sub-region is taken out of production.

#### (2) Uniform land saving

Taking cropland out of production at specific locations can go along with social implications [39], for example by affecting the livelihood of farmers, and thus can reproduce and even foster existing spatial inequalities. We therefore simulate a uniform land saving scenario, where regional policies counteract to balance potentially emerging spatial disparities and a marginalization of people living and working at locations with unfavorable environmental conditions and resulting low yield potentials. Thereby, current cropping patterns are maintained and the cultivated area of each crop category is reduced uniformly across the cropland within a sub-region to avoid a spatial concentration of agricultural production at only the most productive locations for each crop category. Contrary to the BLS, spatial differences in biophysical yield potentials within a sub-region are not taken into account, and cropland of each crop category is taken out of production at low- as well as high-yielding locations (Fig 1). The result can thus serve as a lower bound and rather a conservative estimate of the land saving potential.

The algorithm to simulate ULS calculates for each crop category the attainable production surplus under potential yields on current cropland within each sub-region. With the area weighted mean potential yield in the sub-region and the current statistical production target, this crop-specific surplus can be converted into an area surplus, which represents the land saving potential that can be realized for each crop category when cropland is uniformly reduced at low- and high-yielding locations of each crop category by the share of the area surplus on the current statistical cropland area.

For both, the BLS and ULS strategy, the land saving induced changes in agricultural productivity and cropland requirements are integrated in DART-BIO to assess the effects of land saving on prices, production and trade.

#### (3) Socio-economic land saving

Within the third strategy, land saving is additionally driven by regional socio-economic conditions. We assume that land saving is implemented in a commercialized agricultural framework and thus in a way that simultaneously aims to maximize the value of production and accordingly the attainable profit of crop producers. Therefore, current cropping patterns are reorganized to more profitable ones, so that the most profitable crop categories are allocated at the highest yielding locations within each sub-region.

To simulate this land saving strategy, we couple the biophysical yield potentials of PROMET with DART-BIO, based on the coupling approach from Mauser et al. [28]. It uses the marginal profitability of crop categories within a sub-region together with the spatially heterogeneous biophysical yield potentials to create cropping patterns that maximize the attainable marginal profit. This is achieved by re-allocating the most profitable crop categories at the most productive (considered as high yielding) locations within a sub-region (Fig 1), accounting for the profit-maximization behavior of crop producers. However, we assume that the emergence of monocultures is disrupted by risk aversion of farmers, and crop rotation is nonetheless practiced. Thus, the coupling algorithm allocates a crop mix at each location which reflects the ratio of profitability between the crops, determined by their marginal profitability and yield potential (S5 Appendix) [28]. For the application within this study, the established coupling approach is extended so that land saving potentials can be evaluated: By successively accumulating the attainable production under potential yields and optimized cropping patterns, the (re-)allocation of a crop category is disrupted as soon as its statistical reference production is reached on the already (re-)allocated cropland. We furthermore modified the coupling approach to allow expansion of specific crop categories into already saved cropland of other crop categories within the sub-region, if an increase of current cropland is necessary to sustain reference production under the new, profit-maximized cropland allocation. This can occur when less profitable crop categories are shifted to less productive locations, where the attainable yields are lower than under current cropping patterns and thus more cropland is required for current production. Nevertheless, an overall expansion of cropland into currently uncultivated land is not possible.

The marginal profitability of a crop category depends, inter alia, on the productivity of land as a primary production factor in relation to the other factor inputs labor and capital. Thus, within the land saving strategy assumed changes in cropland and cropping patterns entail changes of land input and consequently affect the price of land and indirectly the profitability of crop allocation. These feedbacks are taken into account within the SLS by coupling the models iteratively: As for the two other strategies, the effects of the profit-maximized land saving on cropland requirements and associated changes in agricultural productivity are fed back into DART-BIO, resulting in changes of production, demand, trade and crop prices. These changes in turn alter the marginal profitability of crop categories and thus affect the profit-maximized cropping patterns, which, contrary to the BLS and ULS, changes the resulting land saving potentials. Within our iterative coupling approach, the induced changes of marginal profitability therefore again enter the socio-economic land saving algorithm, and new cropping patterns and land saving potentials are simulated. The iteration is carried out until a stable crop-allocation is established (S5 Appendix). To focus on the aim of our study to assess the land saving potential under current production patterns, changes in agricultural production resulting from the economic model are not fed back into the socio-economic land saving algorithm. This moreover allows us to be consistent in our production targets across all three land saving strategies and thus maintain comparability between the strategies.

The resulting SLS potential takes changes in current cropping patterns due to more profit-oriented cropping decisions of crop producers into account, and thereby considers the feedbacks between agricultural intensification, changing land requirements and agricultural markets in terms of prices, production and trade, which are key influencing variables of land use decisions [38].

## Results

### Land saving potentials

**Fig 2:**
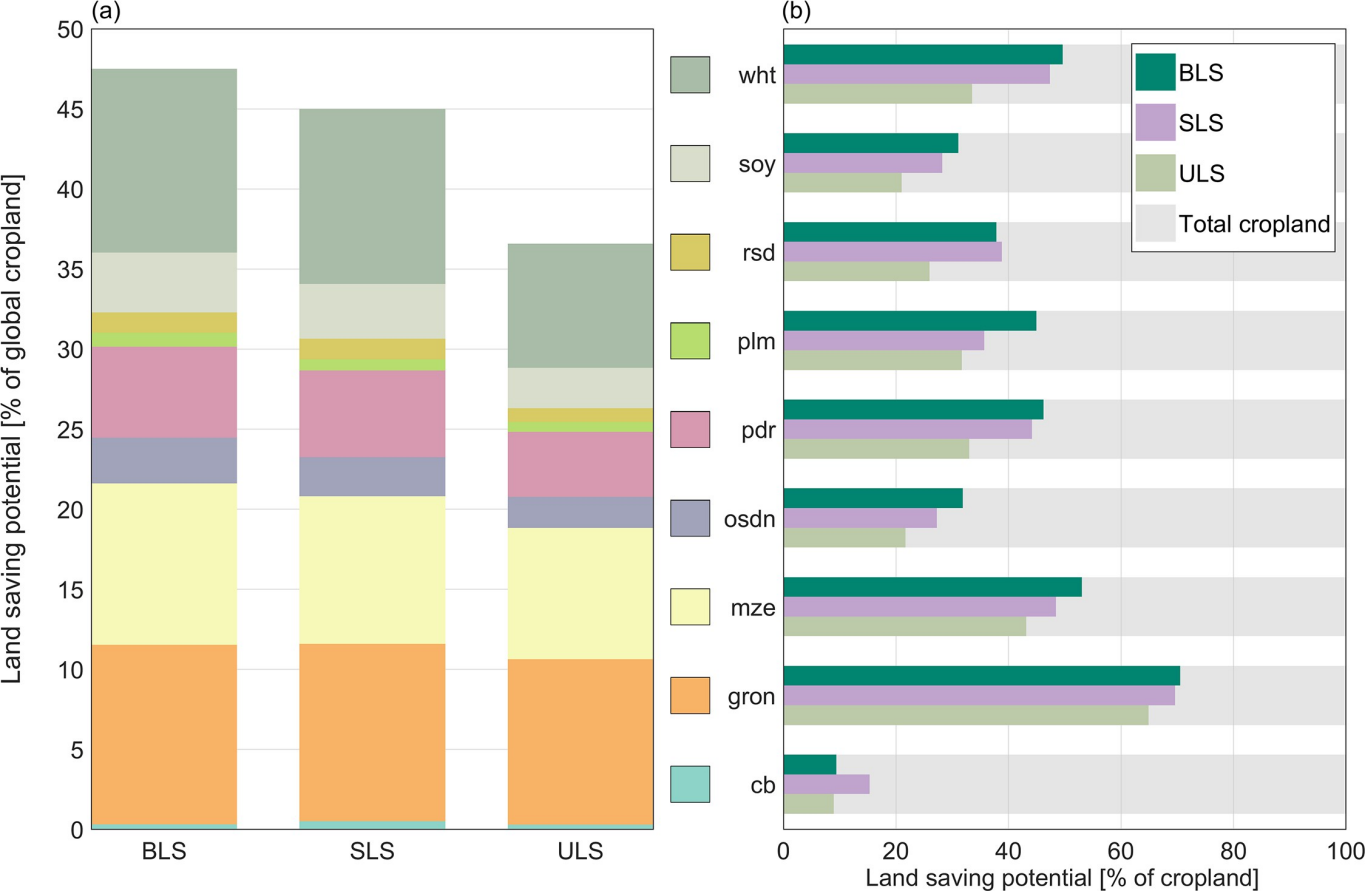
Global land saving potentials. The land saving potential [%] describes the percentage share of cropland that could be taken out of production on the total global cropland area. (a) Global land saving potential for the three different land saving strategies, biophysical land saving (BLS), socio-economic land saving (SLS) and uniform land saving (ULS), further disaggregated into crop categories. (b) Global land saving potential for each crop category as a percentage share of the crop-specific global cropland. For the different crop categories, the following abbreviations are used (Table in S2 Appendix): cb: sugar cane & sugar beet; gron: rest of cereal grains; mze: maize; osdn: rest of oil seeds; pdr: paddy rice; plm: oil palm; rsd: rapeseed; soy: soy; wht: wheat.

Globally, between 37% and 48% of currently cultivated cropland could be taken out of production, depending on the implemented land saving strategy (Fig 2). The largest land saving potentials can be realized under BLS, when crop production is focused on high-yielding cropland. With a profit-optimized allocation and land saving strategy (SLS), this biophysical potential is only slightly reduced, still allowing to save 45% of current cropland. Under the assumption of a ULS strategy, the biophysical land saving potential decreases by 21 percentage points (pp) against the BLS, resulting in a global land saving potential of 37%.

The potential to save cropland varies between different crop categories (Fig 2): Regardless of the strategy, the largest land saving potentials can be achieved for the crop category ‘rest of cereal grains’, which includes crops like sorghum and millet, mainly resulting from large yield gaps in their main growing regions (Figure E and F in S7 Appendix) in Sub-Saharan Africa, India, or the Middle East and Northern Africa. Globally, 65% (ULS) to 70% (BLS, SLS) of current cropland cultivated with ‘rest of cereal grains’ could be taken out of production. Maize, wheat, and paddy rice show medium global land saving potentials between 43% and 53%, 34% to 50%, and 33% to 46%, respectively, depending on the land saving strategy. The lowest land saving potential can be realized for sugar cane and sugar beet, crops that are often cultivated with a high degree of intensification and thus show rather low yield gaps. Only 9% to 15% of global cropland currently cultivated with sugar cane and sugar beet could be taken out of production. In summary, we see large land saving potentials for typical smallholder crops, which are predominantly cultivated with large yield gaps in developing regions [53], while crops that are already intensively cultivated, and thus show rather low yield gaps, have correspondingly lower land saving potentials.

**Fig 3:**
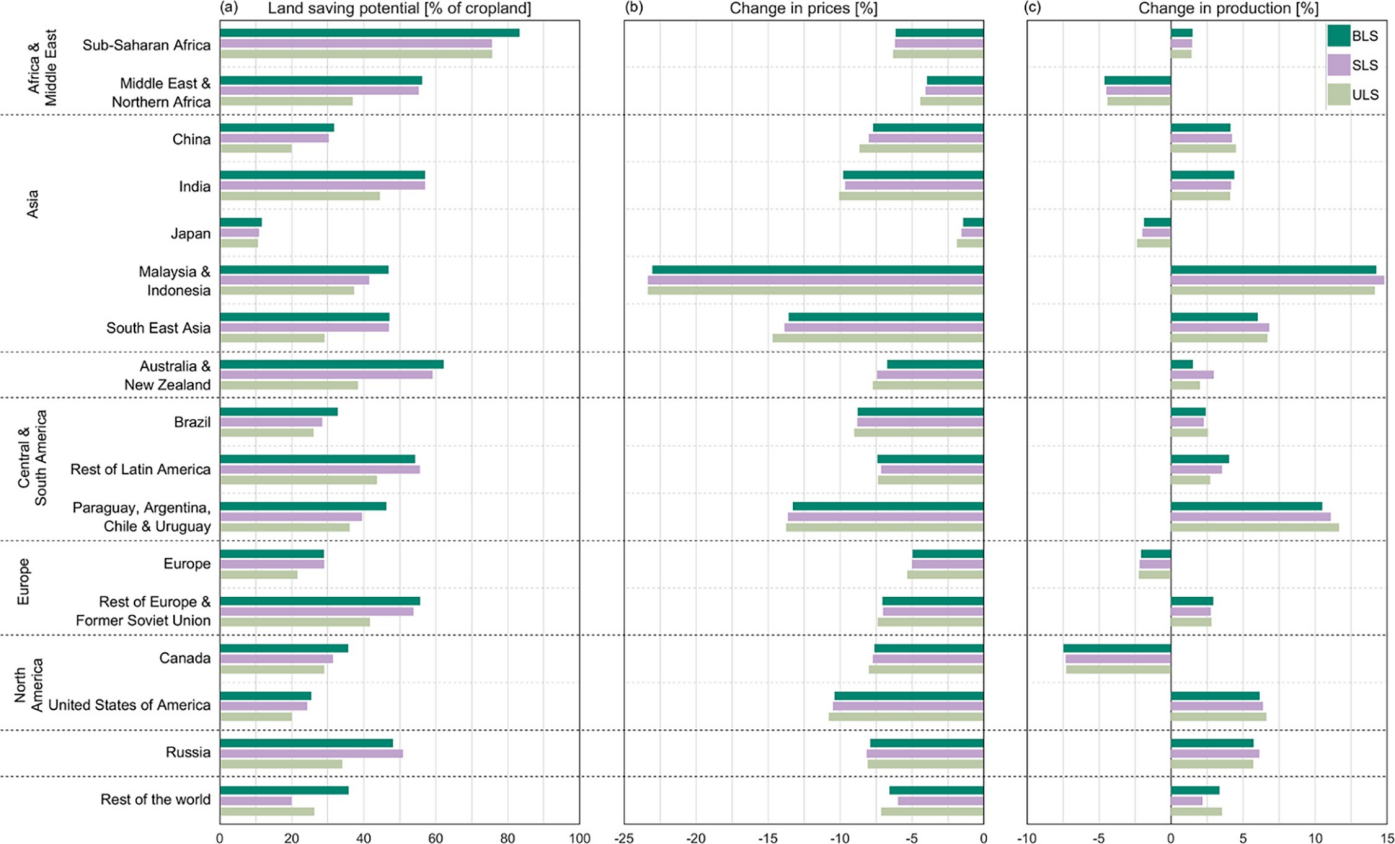
Regional potentials of land saving (a) and resulting regional effects on agricultural markets in terms of changes in crop prices (b) and production (c) for each land saving strategy. Land saving potentials [%] describes the percentage share of cropland that could be taken out of production relative to the total regional cropland across all crop categories. The relative changes in prices and production [%] refer to a baseline without the implementation of land saving.

Regionally, the largest land saving potentials across all strategies can be found in Sub-Saharan Africa, where 76% to 83% of current cropland could be saved. Also, India (44% to 57%), Rest of Latin America (44% to 56%), and Former Soviet Union (42% to 56%) show high land saving potentials across all three strategies. With the BLS and SLS strategy, land saving potentials above 50% can additionally be achieved in Australia and New Zealand, with 62% and 59%, respectively, and both land saving strategies enable to take 55% of current cropland out of production in the Middle East and Northern Africa (Fig 3). These six top regions for land saving currently represent 41% of global cropland and account for 56% of the global land saving potential. Strategically sparing low-yielding or unprofitable cropland in these regions by implementing BLS or SLS could thus reduce the global cropland requirement by 25% (SLS) to 27% (BLS). Even uniform land saving across those six regions could save 20% of currently cultivated global cropland.

Despite rather low relative land saving potentials of 20% to 32%, China is the region with the third largest absolute potential for land saving after Sub-Saharan Africa and India, due to its large absolute cropland area. Together, those three regions account for over a third (37%) of current global cropland and the implementation of land saving in those regions could save between 17% (ULS) and 21% (BLS) of global cropland requirement.

The smallest land saving potentials over all three strategies occur in Japan, the United States of America (USA) and Europe, with 11% to 12%, 20% to 24% and 22% to 29%, respectively (Fig 3 and Table A-C in S7 Appendix).

### Impacts on agricultural markets

The implementation of land saving in the economic model causes global crop prices to fall and triggers an increase in global crop production by +2.8% under all land saving strategies. However, while crop prices fall for all crops and in all regions due to the more efficient use of land, the effect of land saving on production as well as the magnitude of price and production change varies strongly between regions (Fig 3).

**Fig 4:**
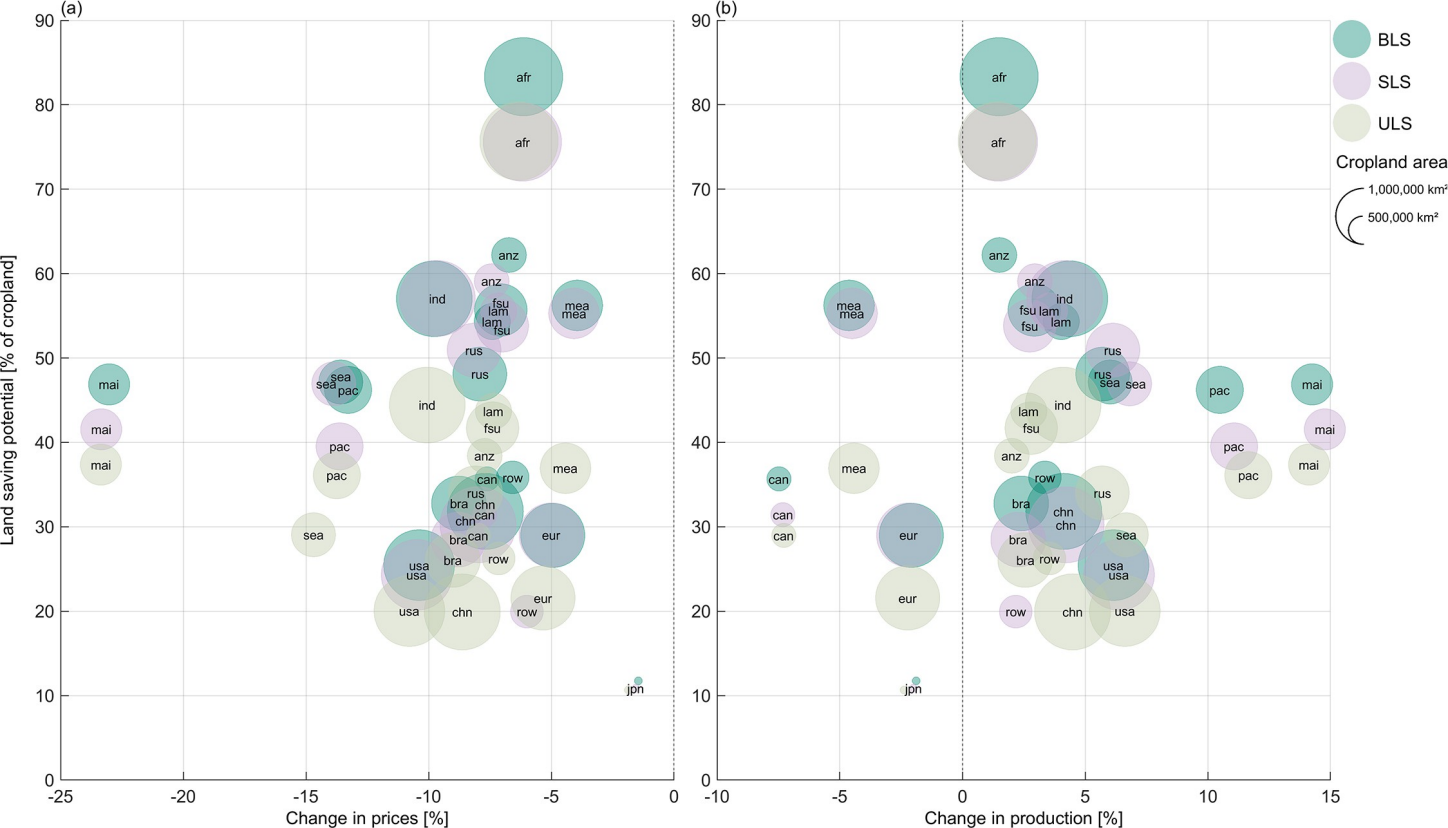
Land saving potentials and their resulting changes in production (a) and prices (b) aggregated over all crop categories for each region. The sizes of the dots reflect current statistical cropland area over all considered crop categories, while the colors of the dots display the different land saving strategies biophysical land saving (BLS), socio-economic land saving (SLS) and uniform land saving (ULS). For the abbreviations and further information on the regions of the analysis, see S3 Appendix.

Our results indicate that the strongest impacts on agricultural markets do not occur in regions with the largest land saving potentials (Fig 4): The largest increases in total crop production can be observed in Malaysia and Indonesia and in Paraguay, Argentina, Chile and Uruguay, which are regions with medium land saving potentials between 36% and 47%. Regional crop production in Malaysia and Indonesia increases by almost +15%, mainly for maize, oil palm and paddy rice, the crops with the largest land saving potential in the region (Figure C in S7 Appendix). As a result, their prices fall by -49% (paddy rice), -36% to -37% (oil palm) and -34% to -38% (maize). Even though the absolute productivity and subsequently output increases for Paraguay, Argentina, Chile and Uruguay are small compared to other regions, the relative increase in output of +10% to +12% compared to a baseline without land saving is large. It mainly results from a rise in the production of soy (+16% to +18%) and wheat (+15% to +16%). Since more than half of the cropland in this region is cultivated with soy, the rather medium land saving potential for soy (32% to 42%) entails a large absolute effect of productivity increase. Soy prices fall accordingly by -16%. In Sub-Saharan Africa, the region with the globally largest land saving potential, the increase in crop production is rather small at +1.4% to +1.5%. Production increases only marginally, mainly for the ‘rest of cereal grains’ (+8% to +10%) and maize (+7% to +8%) and even decreases for wheat by -23% to -30%. In India, on the contrary, where overall land saving potentials are on average across all strategies 25pp lower than in Sub-Saharan Africa, crop production increases by around +4% (+4.1% to +4.5%), mainly resulting from an increase of +17% to +30% in the production of maize, ‘rest of cereal grains’, soy and the ‘rest of oil seeds’, while prices fall by -22% up to -37% (Table D-I in S7 Appendix).

We find that the magnitude of the impact of land saving on production and prices in a region strongly depends on the value share of land in the production costs of crops relative to all other inputs, i.e. labor, capital and intermediate goods such as fertilizer. While the land value shares are roughly in the same order of magnitude for most crops in each region, the differences among regions are large (Table in S1 Appendix). In particular, in Sub-Saharan Africa, the value share of land in the production costs of crops is rather low at 8% to 12%, indicating the relative abundance of land relative to labor and capital in these regions. Hence, a change in this production factor (as it occurs when implementing land saving) has only a minor impact on production outputs. In contrast, highly populated regions such as Malaysia and Indonesia, India and China exhibit high land value shares, as land is a scarce and therefore expensive resource. It amounts to more than 40% in Malaysia and Indonesia, and in India, where globally the second largest land saving potential occurs, the input share in crop production ranges between 19% and 35%. As a result, the effects of our land saving scenario are for example stronger on the Indian agricultural markets compared to those in Sub-Saharan Africa, even though the land saving potential in India is lower.

The results furthermore show that, due to the globalization of agricultural markets, the observed regional impacts of land saving on agricultural markets are not only driven by regional land saving effects, but also by those in spatially distant regions as well as by global markets and crop prices. The Middle East and Northern Africa for example has been a net importer of crops all along. But as the increase in land productivity does not have a large impact on crop production due to a low land value share of 7% to 10%, the falling world market prices within our land saving scenarios lead to a reduction of domestic crop production (-4.4% to -4.6%) and a drastic increase in crop imports (+28% to +29%), mainly wheat and maize from the USA, Russia, and Paraguay, Argentina, Chile and Uruguay. In contrast, in the Rest of Latin America, high potentials to save land and increases in agricultural productivity for soy and maize cause their domestic production to rise by +27% (ULS), +37% (BLS) to +75% (SLS), and +48% (ULS), +56% (SLS) to +65% (BLS), respectively, so that overall crop production increases by +3% to +4%, while net exports of all crops increase by +28% to +50% relative to the baseline. As a consequence, crop consumption rises by +1.3% to +1.5%.

Land saving induced changes in agricultural productivity can thus affect crop import and export in specific regions and consequently lead to a restructuring of trade patterns. This can be observed e.g. when looking at global soy trade under land saving: The high yield potentials for soy in China lead to a reduction of net imports of soy by -11% to -13% and a doubling of domestic soy production (+95% to +108%). This affects the two biggest exporters of agricultural goods, Brazil and the USA. While in Brazil soy exports decline by -22% to -23% and soy production is reduced by -15% to -16%, soy production in the USA decreases by -4% to -6% and net exports are reduced by -5% to -8%. These changing trade patterns are a direct results of the increase in productivity of soybean in China, which led to higher domestic production and lower domestic prices. As a consequence, more soybean is bought domestically and less soybean is imported from Brazil and the USA, which has become relatively more expensive than the domestically produced soybean. The two regions with the highest output increases, Malaysia and Indonesia and Paraguay, Argentina, Chile and Uruguay also exhibit larger exports and smaller imports of agricultural products. Malaysia and Indonesia can increase the exports of palm oil by +21% and decrease maize and soy imports by around -90%. Similarly, Paraguay, Argentina, Chile and Uruguay show higher exports of all agricultural crops, ranging between an increase of +25% for wheat and +12% for soy. In general, we see a reduction of imports of agricultural commodities indicating that more crops are produced domestically.

Overall, we find for all three land saving strategies that the effects on agricultural markets are determined by the interaction of three different effects: Firstly, the magnitude of the land productivity shock is very sensitive to the value share of land in the production function. As it is larger in regions, where land is a scarce and therefore expensive resource, also the impacts of land saving on prices and production are stronger in those land scarce regions, even though the land saving potentials might be smaller than in other regions. A second important factor affecting the economic effects of land saving is the relative relevance of a crop within a region in terms of cropland area: The land productivity shock also depends on the absolute cropland area of the crop that exhibits productivity increase through land saving in a region. So even if the productivity increase for a certain crop is relatively small, large productivity shocks can occur, if the crop accounts for a large proportion of total cropland in the region. Thirdly, the regional economic effects of land saving are also influenced by global market prices and trade patterns and can be displaced to distant regions by trade, as it can be seen in the case of global soy trade or the domestic wheat production in the Middle East and Northern Africa.

### Yield gap closing and strategic land saving

**Fig 5:**
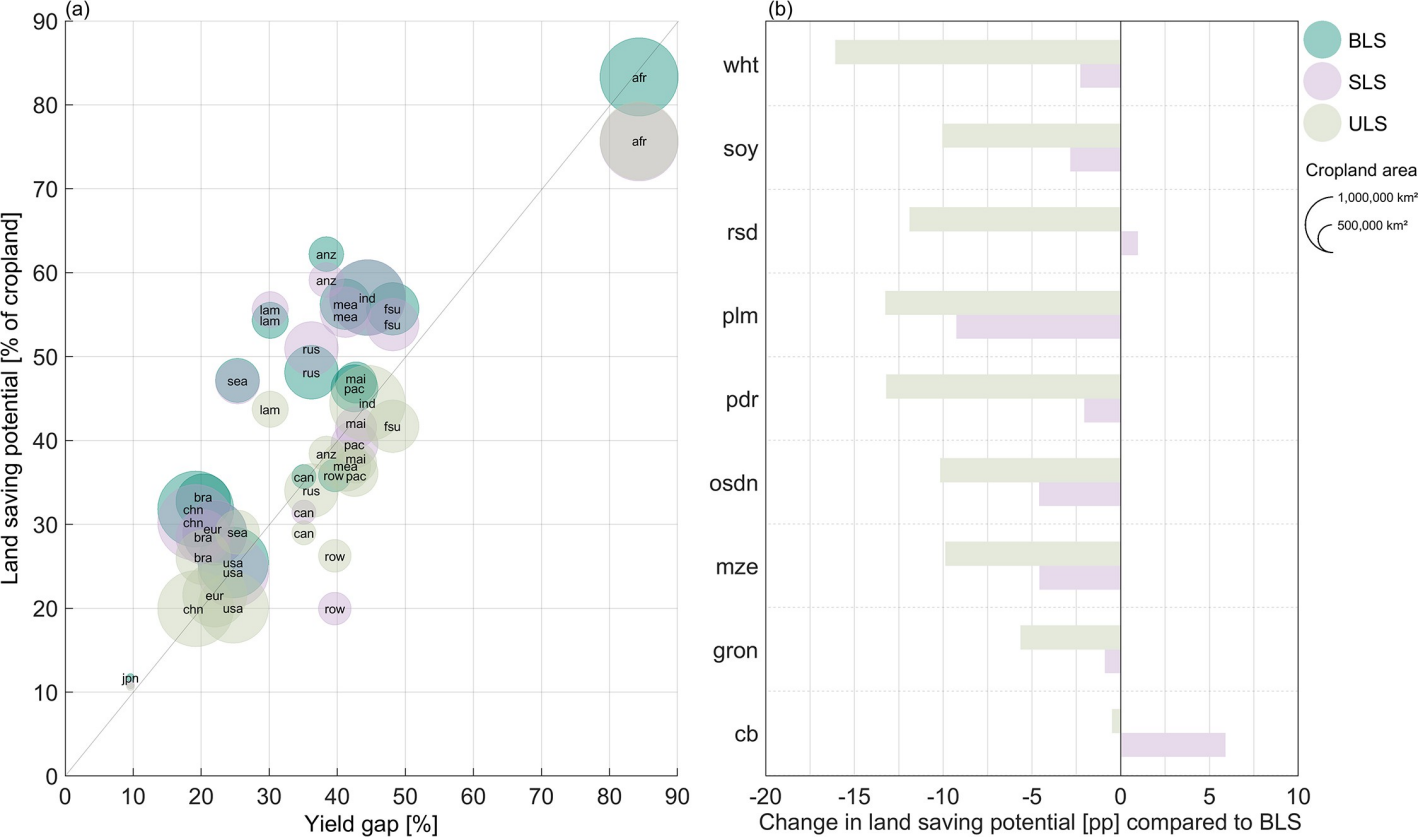
Differences between the land saving strategies in their correlation with current yield gaps and their land saving potential. (a) Regional yield gaps [%] accumulated over all crops and associated regional land saving potential [% of cropland] for biophysical land saving (BLS), socio-economic land saving (SLS) and uniform land saving (ULS). The yield gap is defined as the percentage difference between statistical and potential yields. (b) Change in global land saving potential in percentage points (pp) for each crop category compared to the BLS as upper benchmark for realizable land saving. For the different crop categories, the following abbreviations are used (Table in S2 Appendix): cb: sugar cane & sugar beet; gron: rest of cereal grains; mze: maize; osdn: rest of oil seeds; pdr: paddy rice; plm: oil palm; rsd: rapeseed; soy: soy; wht: wheat. For the abbreviations and further information on the regions of the analysis, see S3 Appendix.

In general, the land saving potential mainly depends on the potential for agricultural intensification and thus the current yield gap. Accordingly, the results show that regions with larger yield gaps tend to have higher land saving potentials (Fig 5). Yet, this correlation varies for the different land saving strategies and is strongest for ULS with a Pearson’s correlation coefficient of +0.93, as land saving is implemented uniformly across sub-region and thus strongly depends on the mean yield gap closing across the whole region. The BLS is focused on high yielding locations, so that locations with lower yields have a lower impact on the land saving potential and accordingly, the correlation coefficient is slightly lower at +0.86. As the SLS potential is moreover influenced by the profitability of crops, the correlation of the land saving potentials with the mean regional yield gaps is lowest for this land saving strategy at +0.75.

In addition, also the spatial implementation of land saving itself, represented by the different strategies for land saving, impacts the realizable cropland reduction. We expected the SLS and ULS potential to be substantially lower than the BLS, as the strategies aim to maximize profit (SLS) or to minimize spatial concentration of land saving (ULS) instead of maximizing the land saving potential by sparing the lowest yielding cropland within each region (BLS). Yet, our results show that, especially between the SLS and the BLS strategy, the differences in realizable land saving potentials are surprisingly low (Table A and B in S7 Appendix). Globally, the land saving potential decreases by -3pp under SLS. However, the resulting cropping patterns differ from the cropping patterns under BLS, as through profit-optimized reallocation and land saving, the relatively less profitable crops within a region are shifted to locations with less optimal growing conditions and accordingly lower yield potentials. This can reduce their land saving potential, while the potential of more profitable crops increases. The strongest effects can be observed for the two cash crops oil palm and sugar beet and sugar cane (Fig 5), looking at their main growing regions. For example, in Malaysia and Indonesia, the main growing region for oil palm, the land saving potential for oil palm decreases by -13pp, as other relatively more profitable crops, such as maize, soy, paddy rice or sugar cane and sugar beet, are allocated to productive locations with high yields and thus oil palm is reallocated to less favorable locations. The overall land saving potential in Malaysia and Indonesia decreases with SLS by -5pp. On the other hand, in Brazil, the main growing region for sugar cane, SLS allows to save 22% instead of 9% (BLS) of current sugar cane cropland in the region. Yet, the land saving potential decreases especially for maize (-17pp), but also for paddy rice (-13pp) and soy (-4pp), so that the overall land saving potential in Brazil declines by -4pp compared to a BLS (Figure D in S7 Appendix).

Due to the assumptions of the strategy, the ULS potential is lower than the BLS potential in all regions and for all crops (Fig 5): Globally, the land saving potential decreases and cropland requirement increases by +11pp, as high- as well as low-yielding cropland is taken out of production. The largest difference can be observed for wheat, where globally -16pp less cropland can be saved than under BLS. This mainly results from reduced land saving potentials under ULS in the main growing regions in Russia, Former Soviet Union, Middle East and Northern Africa and China, where up to -30pp less cropland for wheat cultivation can be taken out of production compared to BLS. Further regions with strong changes are South East Asia and India, mainly resulting from decreased land saving potentials for paddy rice of -20pp and -19pp, respectively (Figure D in S7 Appendix).

The globally small differences in land saving potentials between the three strategies indicate that (1) the large biophysical potential for land saving persists when socio-economic conditions are considered and current cropping patterns change towards a profit-maximized cropland allocation. (2) Even an implementation of land saving to strategically counteract spatial concentration of agricultural production could make a major contribution to reduce current cropland requirements.

## Discussion

### Land saving and the Sustainable Development Goals

Reducing cropland extent by increasing land use efficiency via intensification and optimized land use patterns could contribute to important fields of action of the Sustainable Development Goals [5], such as climate change mitigation (SDG 13) or conservation of terrestrial ecosystems and biodiversity (SDG 15). By reducing global cropland extent, greenhouse gas emissions from fertilized soils as well as the water requirements for irrigation are likely to decrease: Folberth et al. [33] show that reducing the extent of cropland is a main driver of reduced irrigation volume, so that land saving could substantially decrease the water requirements for irrigation, if it is implemented without expanding irrigation. Moreover, especially a reduction in paddy rice cultivation area could contribute to a decline in greenhouse gas emissions [33], as the associated CH_4_ emissions largely contribute to total cropland emissions [20]. We identified large potentials to reduce the cultivated area of paddy rice in its top growing regions, India and South East Asia, by 70% and 51%, respectively, but also in Malaysia and Indonesia (61%) and Sub-Saharan Africa (69%) (Figure A and Table A in S7 Appendix). Due to the large potentials to close yield gaps and reduce cropland, we furthermore assume that, even with an increasing future demand of agricultural commodities, cropland expansion into currently unfarmed land could initially be avoided or substantially reduced [4], contributing to prevent or decrease greenhouse gas emissions induced e.g. by carbon stock decline resulting from the conversion of natural habitats like forests, but also grassland to cropland [25, 54–58]. Moreover, especially in the context of the 2° target of the Paris Agreement [59], there’s a growing interest in land based negative emission technologies to mitigate climate change [60]. By identifying new areas that could potentially be used for this purpose, the assessed land saving potentials reframe the opportunity costs of those technologies and could thus contribute to re-think and re-discuss plant based carbon dioxide removal options like e.g. bioenergy carbon capture and storage [61, 62], but also permanent afforestation and reforestation [63, 64]. An estimation of the carbon sequestration potential of our land saving scenarios carried out with the bookkeeping model of land use emissions BLUE [65] shows that additionally between 31 Gt and 41 Gt carbon, which is equivalent to 114 Gt and 151 Gt CO_2_, respectively, could potentially be sequestered on the saved land by renaturation, assuming a transformation from cropland to the respective potential secondary vegetation (see S8 Appendix).

Our results can furthermore add a quantitative perspective on the land-sparing-potential in the context of the land-sparing vs. land-sharing debate, and serve as a basis for regional research on targeted strategies to reconcile food production and biodiversity conservation [17, 66, 67]. For example, we found large land saving potentials in Sub-Saharan Africa and Central and South America. Both regions have been identified as hotspots, where biodiversity is particularly threatened by future cropland expansion [68]. Regional research on potentials for land saving could thus help to avoid or at least question cropland expansion threatening biodiversity in those regions to meet the increasing demand of agricultural commodities in the future.

On the other hand, as the assessed land saving potentials are based on an intensification of current agricultural production, supposed positive effects of land saving on ecosystems and biodiversity could be reversed by the negative impacts of agricultural intensification on the environment. Increased mechanization based on fossil fuels or the production and generally higher inputs of fertilizer can for example increase greenhouse gas emissions [19, 24, 25, 69], or threaten plant species richness [22], while pesticides, habitat homogenization and the loss of landscape elements for example reduce species diversity [21]. Also increasing irrigation can have negative environmental impacts, such as groundwater depletion [26, 27], or N_2_O emissions [70, 71], but can on the other hand also reduce the overall greenhouse gas emissions of crop production, for example by increasing yields and residue returns and thereby enhancing soil carbon storage [71–73] or preventing further cropland expansion [70]. Thus, the overall environmental effects of land saving strongly depend on how agricultural intensification and the associated management practices regarding e.g. fertilizer application, cropping systems, irrigation or tillage are implemented [24, 72–79]. This highlights the importance of sustainable intensification strategies and technologies [67, 80–82] when discussing land saving and its potential positive contributions to protect ecosystems, reduce greenhouse gas emissions and conserve biodiversity.

In this context, a potential source of uncertainty in our land saving potentials is the assumption that the required economic, technological, and institutional means as well as the societal, cultural and infrastructural framework is given to optimize crop management and realize biophysical yield potentials, for example access to credits, knowledge or inputs like fertilizer [83, 84]. We partly take this limitation into account by not fully closing yield gaps and by assessing the land saving potential for different yield gap closing scenarios (S6 Appendix). However, in this context, it is a potential drawback of our approach that we only partly consider the costs of intensification in our economic model, not taking investment and research & development costs into account. Thus, a complete welfare analysis of the socio-economic impacts of land saving on individual household types is not possible within our study. Yet, from a regional perspective, we see that in particular the regions, where we identify largest land saving potentials, such as Sub-Saharan Africa, India or Former Soviet Union, currently show relatively large yield gaps (Figure F in S7 Appendix), especially for typical smallholder crops such as millet or sorghum. In Sub-Saharan Africa and India, smallholder farms represent around 80% of all farms and operate between 30% and 40% of the agricultural land [85, 86]. Thus, a realization of 80% yield gap closure and accordingly large potentials to reduce current cropland extent needs to be discussed critically: Particularly for less endowed smallholders, overcoming knowledge gaps on best practice and the financial, cultural and legal access to food production resources are main constraining factors for closing yield gaps [84, 87, 88]. Moreover, high costs of agricultural intensification combined with biophysical and socio-economic uncertainties, such as unreliable markets, weather uncertainties, plant pests and diseases or labor shortage, can be an additional barrier for farmers to adopt yield-enhancing technologies [53, 84, 89, 90]. As soon as the risk of additional inputs to close yield gaps being unprofitable is too high, it becomes unlikely that yield gaps are closed [84]. However, technological progress as well as institutional frameworks could help to reduce these uncertainties for farmers, for example information technology to monitor spatial variability of soil nutrients or institutional programs to improve the knowledge of farmers about plant pests and possibilities to control them, or the amount and timing of fertilizer application. Reducing uncertainties changes the economically optimum decision for intensification and could thus encourage farmers to invest and implement measures that close yield gaps and enable land saving [84, 91].

Regarding the large land saving potentials and the large share of smallholders in those regions, it might moreover be challenging to implement land saving without jeopardizing the livelihoods of smallholders [92, 93]. Within the BLS strategy, more than 80% of currently cultivated cropland could be taken out of production in Sub-Saharan Africa and over 50% in India. Even under the less spatially concentrated uniform land saving strategy, still 76% and 44% of cropland could be saved in Sub-Saharan Africa and India, respectively. By taking such large shares of currently cultivated land out of production, many farmers could lose main parts of their income, depending on their income diversity, and also rural population engaged in agriculture, for example as agricultural workers, could be negatively affected. Yet, the saved cropland not necessarily needs to lose its potential to create income: Innovative policies and projects could help to enable a further economic value creation on saved land and co-create environmental benefits, e.g. by economically rewarding carbon sequestration or biodiversity conservation, and support the transition into other livelihoods [93].

Moreover, it is important to mention, that population growth and dietary change are important drivers of future cropland requirements for food production [94] and the effects of climate change will strongly affect agricultural production in terms of its spatial structure and potential productivity [95–98]. As the focus of our study is on the potential contribution of optimized crop and farm management and the effect of strategic crop allocation for land saving, we assess the land saving potential under current climatic and economic conditions. This avoids an overlap with additional effects of different future development paths, such as changes in demand, production patterns and volumes or climate change. However, considering further possible contributions to reduce the pressure on land, like a dietary shift towards increased plant-based diets or food waste reduction [99–104], could help to identify combinations of different measures for reaching a more efficient land use.

### Implications of land saving on food security

While our aggregation to regional households in the economic model does not allow to make any statements about distributional impacts between different household types, a view on crop prices, especially for the staple crops wheat, paddy rice and maize, gives a general idea about potential effects on food security in terms of economic access to food. Given that most poor households in developing countries are net consumers of food [105], the large drop in global agricultural prices through land saving is on one hand likely to increase food security in the short run, as the access to food for poorer households, which spend a large share of their income on staples, improves [106–109]. We find that prices for paddy rice and maize drop by up to -49% and -38%, respectively, especially in Malaysia and Indonesia, India and South East Asia, while prices for wheat fall by up to -27% especially in Russia, China and Paraguay, Argentina, Chile and Uruguay. Also in Sub-Saharan Africa prices for staples drop between -12% and -14%. However, net producers of food do not necessarily benefit from lower prices of agricultural commodities.

On the other hand, studies that looked at the medium run and adaptive responses of supply and demand especially of rural households found positive impacts of global food price shocks that increase food prices, as agricultural wages rise and smallholder farmers benefit [110, 111]. The findings of these studies would imply that the decreasing food prices in our scenarios could negatively affect smallholder farmers and the food security of the rural population. Yet, in our study, the lower prices of agricultural commodities are a result of all farmers, including smallholders, becoming more productive, not a global food price shock. Especially in regions with a large share of smallholders like Sub-Saharan Africa, India or South-East Asia [85, 86, 112], we find an increase in agricultural GDP of +7%, +23% and +44%, respectively. While this increase generally implies higher incomes for farmers, we cannot say whether this is distributed evenly among all farmers and thus whether the access to food is improved.

In terms of domestic production and food sovereignty, we see tendencies in both directions: Falling world market prices can make crop imports cheaper than domestic production and thus regionally reduce food sovereignty, e.g. wheat production in the Middle East and Northern Africa, while an increased productivity via land saving can on the other hand also decrease imports and increase domestic production, such as in Rest of Latin America. In general, our results show a larger specialization towards the widely consumed staple crops instead of increasing import pressure through cheaper crop prices. On average, the smallholder intensive regions become less dependent on food imports or can increase their exports. Sub-Saharan Africa for example can increase its maize exports by +8% and reduce rice imports by -8% and other grain imports by -62%, which make up for 85% of cereal consumption. Wheat imports on the other hand grow by +15%. India can increase its exports of rice, maize and other grains by +43%, +24% and +95%, respectively, while wheat exports decrease by -19%. The latter might by more problematic for India as wheat makes up about 40% of cereal consumption. South East Asia increases its rice exports by +53% and reduce its maize and other grain imports by -62% and -33%, respectively, whereas wheat imports increase by +22% (but only make up 8% of cereal consumption). Thus, wheat seems to be the only cereal where there is an increase in import competition due to the large production increases in regions like the USA. Nevertheless, the productivity increases in the three regions rather point to lower import competition and strengthening of domestic agricultural production.

In summary, land saving can decrease regional food sovereignty and increase dependency on imports on one hand, while on the other hand consumers can benefit from lower prices of agricultural commodities and effectively increase their available income and welfare and thereby potentially improve access to food. Due to the aggregation to regional households in the economic model, the analysis of net impacts on different types of households, such as smallholder households that produce and consume food simultaneously, goes beyond the scope of our study, since we have only one representative household per region. Further research that explicitly takes different households and their income structure into account is necessary to investigate the potential impact and contribution of land saving on food security, especially in smallholder regions.

### Rebound effects and displacement of land saving effects

Earlier studies have shown that intensification does not necessarily induce a decline in cropland area [37, 41], as an increased land use efficiency creates incentives to increase production [113, 114]. We find that globally, land saving triggers an increase in production of all crops compared to statistical reference, while from a regional perspective, particularly land-scarce regions with a high input share of land, such as Malaysia and Indonesia, show large production increases induced by land saving. Since the aim of our study is to assess the economic implications of land saving under current regional production patterns, we restrict available land in the economic model by assuming that saved land is no longer available for crop production. Thus, we cannot capture the full rebound effect, where an increased agricultural production diminishes land saving potentials by taking already saved cropland back into agricultural production or even leads to an expansion of cropland into currently uncultivated land. Nevertheless, our results indicate, in which regions rebound effects would be triggered and thus policies might be required to link an implementation of land saving with cropland reduction.

In our results, we furthermore see how regional economic effects of land saving can impact spatially distant regions due to the global connectedness of regions via trade. The displacement of the induced changes is observable e.g. in the case of soy trade, where a land saving induced productivity increase in China leads to a decrease in soy production in the two main exporting regions USA and Brazil. Moreover, we observed drastic effects in Canada. Here the negative spiral of low land saving potentials as well as land abundance leads to reduction of land use, output and exports. Since production of agricultural products in Canada is now more expensive than in other regions, Canadian exports of soy decrease for example by more than -50%, while the region turns from an exporter to an importer of maize. This demonstrates the importance of a global, integrative framework to identify potentials but also possible risks of land saving induced directly or by the caused economic effects on prices, production and trade, locally as well as in spatially distant places.

## Conclusions

In this paper, we evaluated the land saving potential of different spatial implementation strategies and their effects on agricultural markets in terms of prices, production and trade. The results confirm findings from previous studies on large potentials for cropland reduction by yield gap closing [33, 43]. Similar to Folberth et al. [33], we find that global cropland requirement could be substantially reduced to nearly half of currently cultivated land under a land saving scenario based on a biophysical optimization of land use, while 45% and 37% of current cropland could be saved under land saving strategies that maximize profit or minimize spatial concentration of land saving. The overall small difference in land saving potentials between the three strategies shows that there are large potentials to reduce cropland requirements with strategies that go beyond a purely biophysically driven implementation, accounting for the regional socio-economic framework and potential risks of land saving in terms of spatial marginalization.

**Fig 6:**
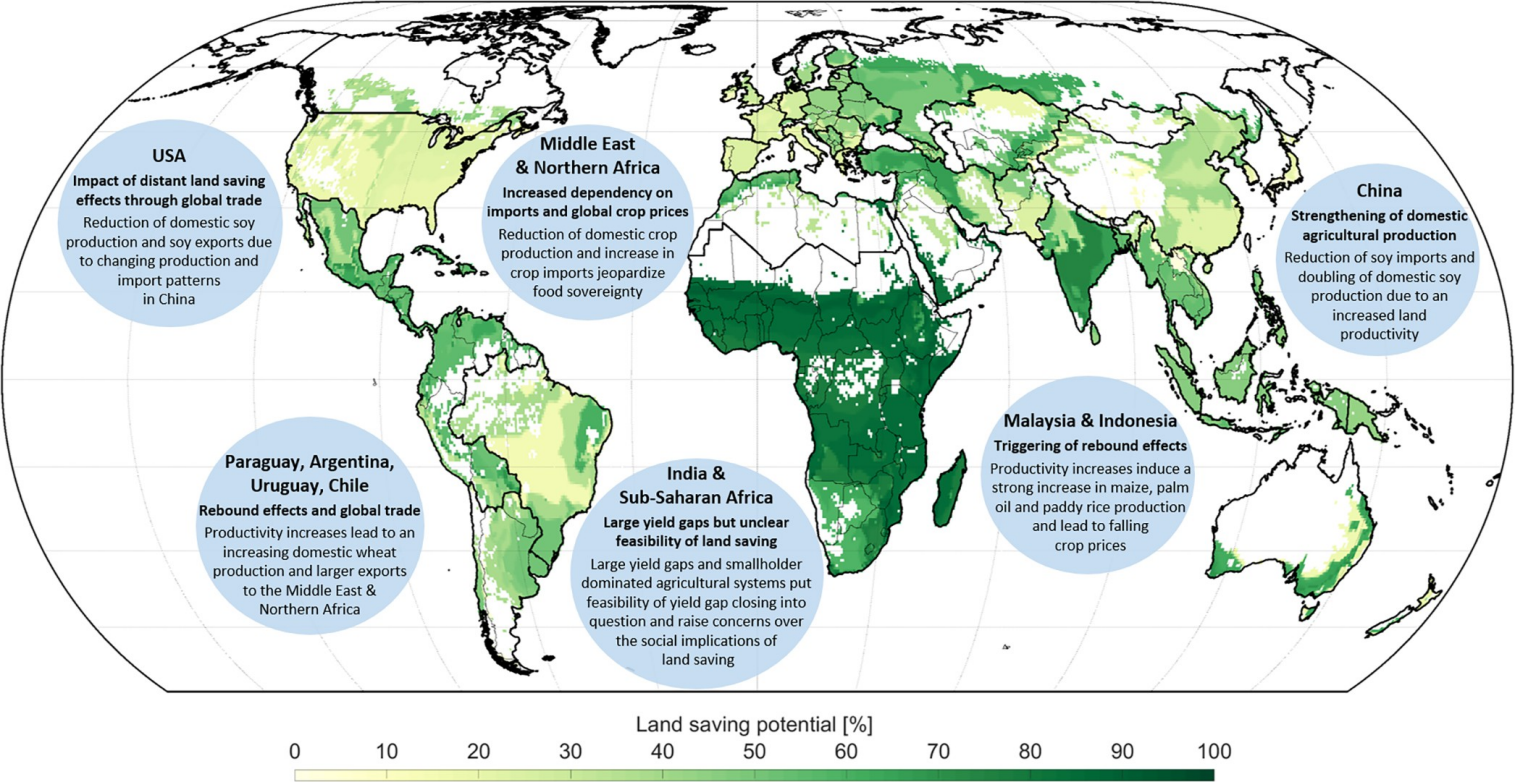
Graphical summary of identified regional effects and implications of land saving. Different identified effects are displayed exemplarily for the most dynamic regions. The underlying global map shows the biophysical land saving potential (BLS) in percentage of total cropland (summed up over all considered crops according to Monfreda et al. [115], see S2 Appendix) for each sub-region. As land saving can only be implemented on current cropland areas, land that is currently not used as cropland is masked out. The thick region lines show the aggregated 17 study regions (see S3 Appendix), while the country boarders are displayed according to the global administrative areas of GADM version 2.8. Reprinted from GADM (https://gadm.org/) under a CC BY license, with permission from GADM, original copyright 2012.

By investigating the effects of land saving on agricultural markets within an integrative approach that links local, regional and global scale, we go beyond biophysical potential evaluations and extend the debate on land saving by adding an interdisciplinary quantitative perspective on impacts on and feedbacks with agricultural markets. The resulting reduction of cropland requirement to 52% to 63% of current global cropland causes crop prices globally to fall for all crop categories, which could have positive impacts on food security. However, the increased efficiency of land use and agricultural production would, on the other hand, cause global crop production to increase by around +2.8%, and thereby partly reduce current land saving potentials. Moreover, we find that the impacts of land saving on agricultural markets are non-linear, so that the strongest changes in prices, production and trade do not generally occur in regions with large cropland reduction potentials. The effects on agricultural markets are mainly determined by the value share of land in the production costs of agricultural goods, the relative relevance of a crop within a region in terms of cropland area and by global market prices and trade. Thus, our study points out the importance of an integrative, global approach as well as the consideration of trade, when land saving and its impacts on agricultural markets are analyzed. The identified regional differences in potential impacts and implications of land saving (Fig 6) moreover show the relevance of taking global-local-global linkages into account and point to a variety of further research questions in the context of land saving, such as occurring rebound effects in the context of global trade, or social implications in different agricultural systems.

Land saving can change the potentials and opportunity costs of further strategies to reconcile food production and environmental conservation, such as afforestation or negative emission technologies to reduce greenhouse gas emissions, or establishing protected areas to protect nature and biodiversity. The results of our study can serve as a starting point to assess the potential for different usages of the freed up land, such as for carbon sequestration or biodiversity conservation, so that the possible contribution of those strategies to approach for example the goals of the Paris Agreement [59] or the post-2020 global biodiversity framework [116], or to reduce trade-offs between different Sustainable Development Goals [5] can quantitatively be re-assessed and re-discussed. Whether land saving can in the end contribute to reaching those goals, however, depends on how agricultural intensification is implemented and the freed up cropland is used. Thus, further research is needed to quantitatively link the land saving potential with the entailed positive and negative environmental implications. Combining our land saving approach with simulation models to estimate carbon fluxes due to land cover conversion [65, 117] as well as investigating the effect and interplay of future scenarios of different drivers [38, 118] on land saving potentials represent interesting perspectives for future research. Overall, an interdisciplinary research framework is necessary to move beyond the theoretical construct of reducing cropland requirements, allowing to investigate measures and mechanisms that can link intensification and land saving without neglecting their social and socio-economic effects and opportunity costs, for example financial compensation and subsidies, or the provision of technology and knowledge. Together with further strategies such as dietary shifts, reduction of food waste and a sustainable intensification, land saving can contribute to reduce the pressure on land resources and play a key role in action on the main global challenges of the 21st century.

## Supporting information

S1 Appendix Model descriptions. (PDF)

S2 Appendix Crops and crop categories. (PDF)

S3 Appendix Spatial structure of the analysis. (PDF)

S4 Appendix Yield potentials. (PDF)

S5 Appendix Coupling approach and socio-economic land saving. (PDF)

S6 Appendix Effect of different yield gap closing scenarios. (PDF)

S7 Appendix Supplementary results. (PDF)

S8 Appendix Carbon sequestration potential of land saving. (PDF)

S1 File Supplementary data: Land saving potentials. (ZIP)
