# Supplementary material for: Global cropland could be almost halved: Assessment of land saving potentials under different strategies and implications for agricultural markets
Source: PLoS One. 2022 Feb 22;17(2):e0263063. doi: 10.1371/journal.pone.0263063 (PMC8863228; doi:10.1371/journal.pone.0263063)
Supplement: S1 Appendix — (PDF) [file pone.0263063.s001.pdf]

S1 Appendix: Model descriptions

For our analysis, we refer to biophysical yield potentials from Mauser et al. 2015 [1], which are simulated with PROMET. The socio-economic context is considered by simulations with DART-BIO. Both models, PROMET and DART-BIO, are briefly described below.

PROMET

PROMET is a biophysical and hydrological land surface process model [2], which has been extended by a biophysical dynamic vegetation component to model crop growth and yield formation [1, 3]. It uses first order physical and physiological principles to determine net primary production and respiration based on approaches from Farquhar et al. [4] and Ball et al. [5], combined with a phenology and a two-layer canopy architecture component of Yin and van Laar [6]. PROMET takes into account the interdependency of net primary production and phenological development, leaf temperature, water availability and environmental conditions including meteorology, CO_2 concentration for C3 and C4 pathways, as well as water and temperature stress. Further details on the PROMET model can be found in Mauser et al. [1].

PROMET has been used in global [1, 7, 8] and regional studies [9]. Moreover, it is used in the Global Gridded Crop Model Initiative (GGCMI) within the Agricultural Model Intercomparison and Improvement Project (AgMIP) [10-12], which is connected to the Inter-Sectoral Impact Model Intercomparison Project (ISIMIP).

The framework and model setup of PROMET for the simulations of the biophysical yield potentials referred to in this study are described in S4 Appendix and Mauser et al. 2015 [1].

DART-BIO

The DART-BIO model is a recursive-dynamic computable general equilibrium (CGE) model of the world economy disaggregated into 23 regions. The model depicts the functioning of regional economies through a system of non-linear equations based on economic theory that are calibrated to an extended version of the Global Trade Analysis Project (GTAP) 9 database [13] with a focus on the production and processing of agricultural commodities and other sectors based on biomass. 40 of the 52 economic sectors in DART-BIO are involved bioeconomy activities. The model features 21 production factors including 18 land types based on the GTAP-AEZs (agro-ecological zones).

In each of the 23 world regions representative consumers interact with producers at commodity and factor markets. Behavior of economic agents is governed by maximization of utility and profit. Consumer maximize their utility according to a Stone-Geary utility function that gives rise to a linear expenditure system. This is calibrated to empirical income and price elasticities for all commodities, which describe the consumers' preferences and govern how demand reacts to income and price changes. Flexible prices and market balance conditions lead to equilibrium of demand and supply on all markets (see [14] for a technical description of the model). Multi-nested constant elasticity of substitution (CES) functions determine sectoral production and ensure imperfect substitution between production factors, which are combined with intermediate inputs through fixed input-output coefficients in Leontief functions. Similarly, bilateral trade is governed by the Armington assumption of imperfect substitutes of domestic and foreign goods and depict with CES functions for imports and CET functions for exports. Consistency of microeconomic behavior and macroeconomic aggregates is ensured through savings driven investment and fixed current account balances (with the United States of America as flexible numeraire

region). The model is solved annually. Long-term dynamics are depicted through updating essential parameters (capital stocks, productivity, labor force and population growth).

Table. Value share of land in production costs [%] for each crop category within each region.

region / crop category	cb	gron	mze	osdn	pdr	plm	rsd	soy	wht
Sub-Saharan Africa	8.40	11.00	10.80	9.80	11.60	12.20	8.20	11.20	7.60
Australia & New Zealand	14.50	9.50	8.70	14.90	11.30	--	14.90	15.20	14.20
Belgium, Netherlands, Luxemburg	9.40	9.50	8.40	20.00	--	--	18.10	--	8.40
Brazil	12.20	11.10	9.90	11.60	10.80	11.70	11.70	10.90	11.00
Canada	11.80	8.30	8.10	10.20	--	--	9.90	9.90	9.20
China	26.80	30.00	23.90	37.50	27.60	38.00	37.70	37.20	23.10
France	10.00	9.90	9.90	10.40	--	--	10.40	11.00	10.20
Former Soviet Union	13.30	16.90	17.70	17.80	3.20	--	17.30	19.30	13.30
Great Britain	12.00	12.60	--	12.80	--	--	12.30	--	11.30
Germany	13.40	14.60	14.70	--	--	--	13.90	--	13.70
India	30.20	28.40	28.60	31.10	34.70	--	32.20	31.80	18.70
Japan	11.00	10.10	--	--	25.30	--	--	8.20	10.80
Rest of Latin America	21.60	21.50	20.00	20.60	20.20	23.80	--	18.70	15.10
Malaysia & Indonesia	38.70	--	44.30	39.30	39.90	37.00	--	40.60	--
Middle East & Northern Africa	7.60	7.70	8.10	8.30	9.60	--	7.80	8.30	6.80
Mediterranean (Italy, Spain, Portugal, Greece, Malta, Cyprus)	24.80	13.20	14.80	12.80	30.30	--	12.60	--	13.80
Paraguay, Argentina, Chile & Uruguay	17.40	15.80	16.20	18.10	18.40	23.00	16.60	17.50	16.60
Rest of Europe (Austria, Estonia, Latvia, Lithuania, Poland, Hungary, Slovakia, Slovenia, Czech Republic, Romania, Bulgaria)	30.20	25.50	26.30	19.10	35.60	--	24.60	23.40	25.80
Rest of the world	30.40	27.50	36.30	33.10	38.00	40.00	25.50	39.10	31.70
Russia	24.60	24.60	24.60	22.30	23.30	--	24.30	21.70	22.80
Scandinavia (Denmark, Finland, Sweden)	15.50	20.80	11.80	10.10	--	--	17.00	--	14.50
South East Asia	30.80	35.00	36.20	28.80	32.50	30.30	15.00	26.20	10.80
United States of America	22.70	18.90	18.50	21.00	18.30	--	21.30	20.00	21.10

The table displays the within DART-BIO assumed value share of land in the production costs of crops relative to all other inputs, i.e. labor, capital and intermediate goods such as fertilizer. For the different crop categories, the following abbreviations are used (for details see also Table in S2 Appendix): cb: sugar cane & sugar beet; gron: rest of cereal grains; mze: maize; osdn: rest of oil seeds; pdr: paddy rice; plm: oil palm; rsd: rapeseed; soy: soy; wht: wheat. For further information on the spatial structure and regions of the analysis, see S3 Appendix.

References

1. Mauser W, Klepper G, Zabel F, Delzeit R, Hank T, Putzenlechner B, et al. Global biomass production potentials exceed expected future demand without the need for cropland expansion. Nat Commun. 2015;6. doi: 10.1038/ncomms9946.
2. Mauser W, Bach H. PROMET - Large scale distributed hydrological modelling to study the impact of climate change on the water flows of mountain watersheds. Journal of Hydrology. 2009;376(3-4):362-77. doi: DOI: 10.1016/j.jhydrol.2009.07.046.
3. Hank TB, Bach H, Mauser W. Using a Remote Sensing-Supported Hydro-Agroecological Model for Field-Scale Simulation of Heterogeneous Crop Growth and Yield: Application for Wheat in Central Europe. Remote Sensing. 2015;7(4):3934-65. doi: 10.3390/rs70403934. PubMed PMID: WOS:000354789300024.
4. Farquhar GD, Caemmerer S, Berry JA. A biochemical model of photosynthetic CO2 assimilation in leaves of C3 species. Planta. 1980;149(1):78-90. doi: 10.1007/BF00386231.
5. Ball JT, Woodrow I, Berry J. A Model Predicting Stomatal Conductance and its Contribution to the Control of Photosynthesis under Different Environmental Conditions. In: Biggins J, editor. Progress in Photosynthesis Research: Springer Netherlands; 1987. p. 221-4.
6. Yin X, van Laar H. Crop Systems Dynamics. An Ecophysiological Simulation Model for Genotype-By-Environment Interactions. Wageningen: Wageningen Academic Publishers; 2005.
7. Zabel F, Delzeit R, Schneider JM, Seppelt R, Mauser W, Václavík T. Global impacts of future cropland expansion and intensification on agricultural markets and biodiversity. Nature Communications. 2019;10(1):2844. doi: 10.1038/s41467-019-10775-z.
8. Jägermeyr J, Robock A, Elliott J, Müller C, Xia L, Khabarov N, et al. A regional nuclear conflict would compromise global food security. Proceedings of the National Academy of Sciences. 2020;117(13):7071. doi: 10.1073/pnas.1919049117.
9. Degife AW, Zabel F, Mauser W. Climate change impacts on potential maize yields in Gambella Region, Ethiopia. Regional Environmental Change. 2021;21(2):60. doi: 10.1007/s10113-021-01773-3.
10. Minoli S, Müller C, Elliott J, Ruane AC, Jägermeyr J, Zabel F, et al. Global Response Patterns of Major Rainfed Crops to Adaptation by Maintaining Current Growing Periods and Irrigation. Earth's Future. 2019;7(12):1464-80. doi: 10.1029/2018EF001130.
11. Franke JA, Müller C, Elliott J, Ruane AC, Jägermeyr J, Snyder A, et al. The GGCMI Phase 2 emulators: global gridded crop model responses to changes in CO2, temperature, water, and nitrogen (version 1.0). Geosci Model Dev. 2020;13(9):3995-4018. doi: 10.5194/gmd-13-3995-2020.
12. Müller C, Franke J, Jägermeyr J, Ruane AC, Elliott J, Moyer E, et al. Global gridded crop model evaluation: benchmarking, skills, deficiencies and implications. Geosci Model Dev. 2017;10:1403-22. doi: 10.5194/gmd-10-1403-2017.
13. Aguiar A, Narayanan B, McDougall R. An Overview of the GTAP 9 Data Base. Journal of Global Economic Analysis. 2016;1(1):181-208. doi: 10.21642/jgea.010103af.
14. Calzadilla A, Delzeit R, Klepper G. DART-BIO: Modelling the interplay of food, feed and fuels in a global CGE model. Kiel, Germany: Kiel Institute for the World Economy, 2014.
