# Supplementary material for: Global cropland could be almost halved: Assessment of land saving potentials under different strategies and implications for agricultural markets
Source: PLoS One. 2022 Feb 22;17(2):e0263063. doi: 10.1371/journal.pone.0263063 (PMC8863228; doi:10.1371/journal.pone.0263063)
Supplement: S2 Appendix — (PDF) [file pone.0263063.s002.pdf]

## S2 Appendix. Crops and crop categories

Within our study, we consider 15 globally important crops with respect to area and economic relevance, as they represent 70% of global cropland area and 65% of global crop production according to FAOSTAT (all listed crops, average for 1981-2010 [1]). We include staple crops of global importance, such as maize, wheat, rice and soy, that provide 2/3 of global calorie production, but also consider more regionally important food crops, such as millet or cassava. Furthermore, we capture the main bioenergy crops, such as oil palm, maize, soy, sugarcane and rapeseed, to capture the trends in political support of biofuels. To be consistent with the representation of crops in the economic model, the 15 crops are aggregated to 9 crop categories that are considered in DART-BIO (see Table below).

**Table. Considered crops for simulations of biophysical yield potentials with PROMET and their grouping to crop categories considered in DART-BIO.**

| Crops | Crop Category | |
|---|---|---|
| Sugar cane<br>Sugar beet | cb | Sugar cane & sugar beet |
| Barley<br>Millet<br>Rye<br>Sorghum | gron | Rest of cereal grains |
| Maize | mze | Maize |
| Groundnut<br>Sunflower | osdn | Rest of oil seeds |
| Paddy rice | pdr | Paddy rice |
| Oil palm | plm | Oil palm fruit |
| Rapeseed | rsd | Rapeseed |
| Soy | soy | Soybean |
| Summer wheat<br>Winter wheat | wht | Wheat |

## References

1. FAOSTAT. http://www.fao.org/faostat/en/#data/QC. 2019.
