# Supplementary material for: Global cropland could be almost halved: Assessment of land saving potentials under different strategies and implications for agricultural markets
Source: PLoS One. 2022 Feb 22;17(2):e0263063. doi: 10.1371/journal.pone.0263063 (PMC8863228; doi:10.1371/journal.pone.0263063)
Supplement: S3 Appendix — (PDF) [file pone.0263063.s003.pdf]

S3 Appendix: Spatial structure of the analysis

Our global analysis is structured in 23 regions, that are again divided by Agro-Ecological Zones (AEZ) [1] into sub-regions, resulting globally in 216 sub-regions. Subdividing regions into AEZ enables to better assess the sectoral competition for land. The different AEZ are defined by the length of the growing period (0 to 59 days, 60 to 119 days, 120 to 179 days, 180 to 239 days, 240 to 299 days and more than 300 days) and the climatic zone (tropical, temperate and boreal), described by absolute minimum temperature and Growing Degree Days [2]. To ensure a sufficient number of locations for a robust assessment of the land saving potential, we excluded sub-regions with less than 10 sample locations. As a result, 139 sub-regions, representing 95% of global cropland and crop production of the considered crop categories, are considered within our study. Land saving potentials and impacts on agricultural markets are investigated at sub-region level. For the evaluation and discussion of our results, we aggregated the regions BEN (Belgium, Netherlands, Luxemburg), FRA (France), GBR (Great Britain), GER (Germany), MED (Italy, Spain, Portugal, Greece, Malta, Cyprus), SCA (Denmark, Finland, Sweden), and REU (Austria, Estonia, Latvia, Lithuania, Poland, Hungary, Slovakia, Slovenia, Czech Republic, Romania, Bulgaria) to the Region Europe (EUR), resulting in a total of 17 study regions globally (see Figure A).

References

1. IIASA/FAO. Global Agro-ecological Zones (GAEZ v3.0). In: IIASA L, Austria and FAO, Rome, Italy, editor. 2012.
2. Ramankutty N, Hertel T, Lee H-L. Global Land Use and Land Cover Data for Integrated Assessment Modeling. Purdue University, West Lafeyette, Indiana (www gtap agecon purdue edu/resources/resdisplay asp. 2004.

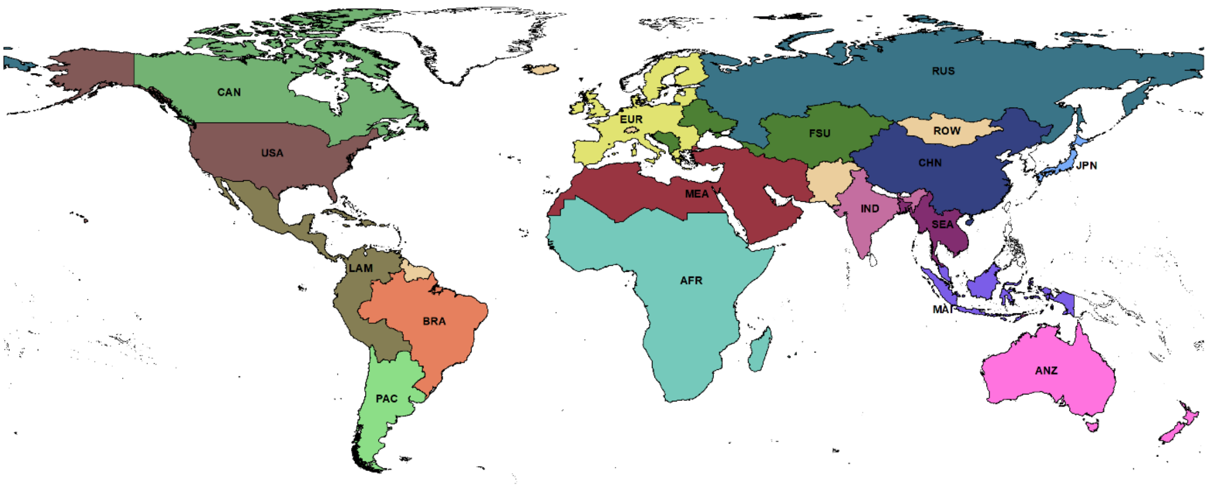

Figure A. Region mapping of the 17 study regions. AFR (Sub-Saharan Africa), ANZ (Australia, New Zealand), BRA (Brazil), CAN (Canada), CHN (China), EUR (Austria, Belgium, Bulgaria, Czech Republic, Cyprus, Denmark, Estonia, France, Finland, Germany, Great Britain, Greece, Hungary, Italy, Latvia, Lithuania, Luxemburg, Malta, Netherlands, Poland, Portugal, Romania, Slovakia, Slovenia, Spain, Sweden), FSU (Rest of Former Soviet Union and Rest of Europe), IND (India), JPN (Japan), LAM (Rest of Latin America), MAI (Malaysia, Indonesia), MEA (Middle East and Northern Africa), PAC (Paraguay, Argentina, Chile, Uruguay), ROW (Rest of the World), RUS (Russia), SEA (South East Asia: Cambodia, Bangladesh, Laos, Myanmar, Thailand, Vietnam), USA (United States of America). The region lines show the aggregated country boarders according to the global administrative areas of GADM version 2.8. Reprinted from GADM (https://gadm.org/) under a CC BY license, with permission from GADM, original copyright 2012.
