# Supplementary material for: Global cropland could be almost halved: Assessment of land saving potentials under different strategies and implications for agricultural markets
Source: PLoS One. 2022 Feb 22;17(2):e0263063. doi: 10.1371/journal.pone.0263063 (PMC8863228; doi:10.1371/journal.pone.0263063)
Supplement: S4 Appendix — (PDF) [file pone.0263063.s004.pdf]

S4 Appendix: Yield potentials

Biophysical yield potentials

A global dataset of biophysical yield potentials for the 15 crops included in this study is provided by Mauser et al. [1]. The potential yields are based on simulations with PROMET on 246,000 representative sample locations on the agriculturally suitable area [2]. Crop growth is simulated hourly for 30 years (1981 to 2010), using high-resolution (30 arcsec) global data on climate from the output of the general circulation model ECHAM5 [3], soil data obtained from the Harmonized World Soil Database [4] and topography derived from the Shuttle Radar Topography Mission [5]. The yield potentials include optimal crop management considering nutrient supply, sowing and harvest dates, as well as realizing multiple harvests and no harvest losses due to pests and diseases. For the simulation of irrigated yield potentials, we furthermore assume that no water stress occurs. The yields are aggregated to 30-year means to avoid bias from selecting a single year, and to crop categories to be consistent with the crop representation in DART-BIO for the model coupling (for details on the sampling approach, input data and model setup see Mauser et al. [1]).

Mauser et al. [1] show that the conducted PROMET simulations provide similar results to existing studies on biophysical production potentials. For example, compared with the FAO-GAEZ [6] global simulations of potential yields and production, significantly similar distributions, means and standard-deviations occur.

Yield gap closing

Biophysical yield potentials are helpful to explore natural potentials and limits as upper benchmarks. However, to account for several constraints limiting the realization of potential yields, such as (socio-)economic or technological factors [7, 8], we do not assume full yield gap closing. In literature, it is often referred to 80% of the potential

yield being considered as an ‘attainable yield potential’ [8-10]. However, assuming a proportion of the biophysical yield potentials would lead to yields that solely depend on the current biophysical production potentials (and thus environmental conditions), while current yield gaps, that strongly depend on the socio-economic conditions, are neglected and regional differences in current yields are not taken into account. We thus decided to assume that yield gaps are closed by 80%, leading to yields that depend on the biophysical yield potential but also on currently achieved yields and yield gaps. This approach was also evaluated as realistic and consistent by stakeholders within a co-design process for scenario-development [11].Therefore, we calculate the mean yield gap for each crop category within a sub-region as the difference between the area weighted mean potential yield under current irrigation and cropping patterns [12] and our statistical reference yield from the GTAP 9 database [13]. Closing this mean yield gap by 80% enables to calculate a mean potential yield share for the sub-region, that is applied to the modelled potential yields at each location to simulate an 80% yield gap closing scenario. If the mean yield gap of a crop category is negative, so that current statistical yields of a crop exceed the simulated potential yields, we refer to the current statistical yields of the crop and do not close the yield gap.

To evaluate the impact of yield gap closing on the land saving potential, we simulated different scenarios from 50% to 100% yield gap closing. The effect on the global land saving potential is shown in S6 Appendix.

References

1. Mauser W, Klepper G, Zabel F, Delzeit R, Hank T, Putzenlechner B, et al. Global biomass production potentials exceed expected future demand without the need for cropland expansion. Nat Commun. 2015;6. doi: 10.1038/ncomms9946.
2. Zabel F, Putzenlechner B, Mauser W. Global agricultural land resources--a high resolution suitability evaluation and its perspectives until 2100 under climate change conditions. PLoS One. 2014;9(9):e107522. Epub 2014/09/18. doi: 10.1371/journal.pone.0107522. PubMed PMID: 25229634; PubMed Central PMCID: PMCPMC4167994.
3. Jungclaus JH, Keenlyside N, Botzet M, Haak H, Luo JJ, Latif M, et al. Ocean Circulation and Tropical Variability in the Coupled Model ECHAM5/MPI-OM. Journal of Climate. 2006;19(16):3952-72. doi: 10.1175/JCLI3827.1.
4. FAO, IIASA, ISRIC, ISSCAS, JRC. Harmonized World Soil Database (version 1.2). FAO, Rome, Italy and IIASA, Laxenburg, Austria2012.
5. Farr TG, Rosen PA, Caro E, Crippen R, Duren R, Hensley S, et al. The Shuttle Radar Topography Mission. Reviews of Geophysics. 2007;45(2):RG2004. doi: 10.1029/2005RG000183.
6. Fischer G, Nachtergaele F, Prieler S, Teixeira E, Toth G, Velthuizen H, et al. Global Agro-Ecological Zones (GAEZ v3.0) - Model Documentation2012.
7. Jiren TS, Dorresteijn I, Schultner J, Fischer J. The governance of land use strategies: Institutional and social dimensions of land sparing and land sharing. Conserv Lett. 2018;11(3):e12429. Epub 2018/07/24. doi: 10.1111/conl.12429. PubMed PMID: 30034527; PubMed Central PMCID: PMCPMC6049885.
8. Lobell DB, Cassman KG, Field CB. Crop Yield Gaps: Their Importance, Magnitudes, and Causes. Annual Review of Environment and Resources. 2009;34(1):179-204. doi: doi:10.1146/annurev.environ.041008.093740.
9. Cassman KG, Dobermann A, Walters DT, Yang H. Meeting Cereal Demand While Protecting Natural Resources and Improving Environmental Quality. Annual Review of Environment and Resources. 2003;28(1):315-58. doi: 10.1146/annurev.energy.28.040202.122858.
10. van Ittersum MK, Cassman KG, Grassini P, Wolf J, Tittonell P, Hochman Z. Yield gap analysis with local to global relevance - A review. Field Crops Research. 2013;143(0):4-17. doi: 10.1016/j.fcr.2012.09.009.
11. Delzeit R, Heimann T, Schuenemann F, Söder M, Zabel F, Hosseini M. Scenarios for an impact assessment of global bioeconomy strategies: Results from a co-design process. Research in Globalization. 2021;3:100060. doi: 10.1016/j.resglo.2021.100060.
12. Portmann FT, Siebert S, Döll P. MIRCA2000—Global monthly irrigated and rainfed crop areas around the year 2000: A new high-resolution data set for agricultural and hydrological modeling. Global Biogeochemical Cycles. 2010;24(1):n/a-n/a. doi: 10.1029/2008GB003435.
13. Aguiar A, Narayanan B, McDougall R. An Overview of the GTAP 9 Data Base. Journal of Global Economic Analysis. 2016;1(1):181-208. doi: 10.21642/jgea.010103af.
