# Supplementary material for: Global cropland could be almost halved: Assessment of land saving potentials under different strategies and implications for agricultural markets
Source: PLoS One. 2022 Feb 22;17(2):e0263063. doi: 10.1371/journal.pone.0263063 (PMC8863228; doi:10.1371/journal.pone.0263063)
Supplement: S5 Appendix — (PDF) [file pone.0263063.s005.pdf]

S5 Appendix: Coupling approach and socio-economic land saving

Marginal Profit Functions

For the coupling, DART-BIO provides sub-region and crop-specific marginal profit functions that are derived from the market equilibrium and depend i.a. on the productivity of land in relation to other factor inputs (capital, labor, energy). They determine the achievable profit for allocating a certain crop category on an additional unit of land as a function of the already allocated area to this crop category. The attainable marginal profit [$/hectare (ha)] is highest for the first cultivated hectare of a crop category and decreases, the more cropland is allocated, until it approaches zero when current cropland area of the crop is reached (Figure A). Within a sub-region, the marginal profit functions can rank the crop categories according to the profitability of allocating an additional unit of its cropland. For details on how marginal profit functions are determined, see Mauser et al. [1].

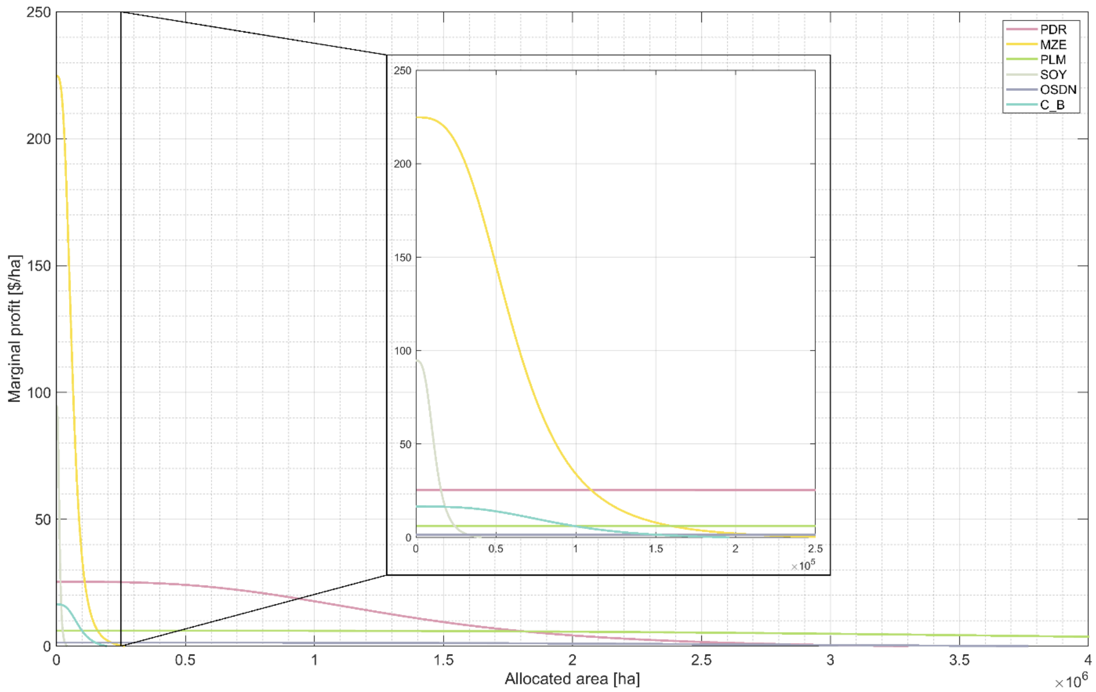

Figure A. Exemplary marginal profit function for the sub-region in AEZ 6 in Malaysia and Indonesia. The x-axis displays the allocatable cropland [hectare (ha)] for each crop category within the sub-region, while the y-axis shows the attainable marginal profit [$/ha] for the allocation of one additional hectare cropland. Marginal profit functions are attainable for all crop categories cultivated within the sub-region.

Coupling PROMET and DART-BIO

Based on the marginal profit functions from DART-BIO and the resulting marginal profitability per production unit [$/t], we can calculate the potential marginal profit per hectare [$/ha] attainable under the potential yields derived from PROMET. It defines the attainable marginal profit by allocating one unit of cropland of a specific crop category. The potential marginal profit per hectare [$/ha] differs between the sample locations within a sub-region due to spatially differing environmental conditions and thus biophysical yield potentials. A maximum achievable total potential profit [$] could be derived by allocating the crop category with the highest potential marginal profit per hectare at each location. However, to account for risk aversion of farmers and the implementation of crop rotation, all profitable crops at a location are allocated, with their cropland ratio reflecting the ratio of the potential marginal profits per hectare. This results in profit-maximized but diversified cropping patterns at each location. Within our coupling algorithm, cropland is then allocated at the most profitable location with the highest achievable total potential profit [$]. Since the attainable marginal profit per hectare changes with the ratio of allocated and total cultivated area of a crop category (Figure A), also the achievable total potential profit at the remaining locations changes after the allocation of cropland at the most profitable location. Thus, the cropland allocation and the thereupon calculated achievable total profit needs to be recalculated for all remaining locations after each allocation at the next most profitable location. These steps are sequentially repeated until current statistical production is reached for all crop categories within the sub-region (for details see Mauser et al. [1]).

When current cropping patterns change to more profitable ones, it is possible that less profitable crops are reallocated to locations with relatively lower production potential, resulting in a larger cropland requirement compared to current statistics for those crops. However, if the statistical acreage has been reached, the marginal profitability

of additional cropland allocation is not yet defined so that the attainable marginal profit is zero, and thus the crop is by definition no longer profitable to allocate. To maintain current crop production, we assume that the crop is nonetheless allocated by a fixed area share, determined by the number of crops that can be allocated at each location. Thus, we allow the expansion of cropland into saved cropland of other crops, if it is necessary to maintain current statistical production, and as long as current statistical cropland over all crops is not expanded. If within a sub-region, profit-maximized reallocation and the resulting cropping patterns are not able to meet the current statistical production targets, we assume that current cropping patterns are maintained and land saving is not implementable within this sub-region.

Iteration

As soon as current statistical production is reached, the allocation is stopped, and the resulting new cropland requirements for each crop category are fed back into DART-BIO (Figure B). Even though the total production of each crop category remains constant, the decreased cropland requirements change the productivity of land as primary production factor and thus affect the marginal profitability of crops, leading to new marginal profit functions. Thus, for the SLS, those changes are again fed back and serve as input for the coupling approach with the socio-economic land saving algorithm, resulting in new profit-optimized cropping patterns and land saving potentials. The iteration hence allows to account for the interplay of land use decision making and resulting cropping patterns and the socio-economic context (demand, prices, trade flows). It is carried out until a stable crop-allocation is established in all sub-regions, which is defined as changes in required cropland and resulting cropland productivity between two iteration-steps are below 1% (Figure C). For this evaluation, we include all crops that are sufficiently relevant within their sub-region, defined by their cropland share on total cropland area in the sub-region and their absolute

cropland area that needs to be greater than 1% and 250 hectares, respectively, and for which land is a relevant production factor and thus the proportion of land in factor input is above 5%.

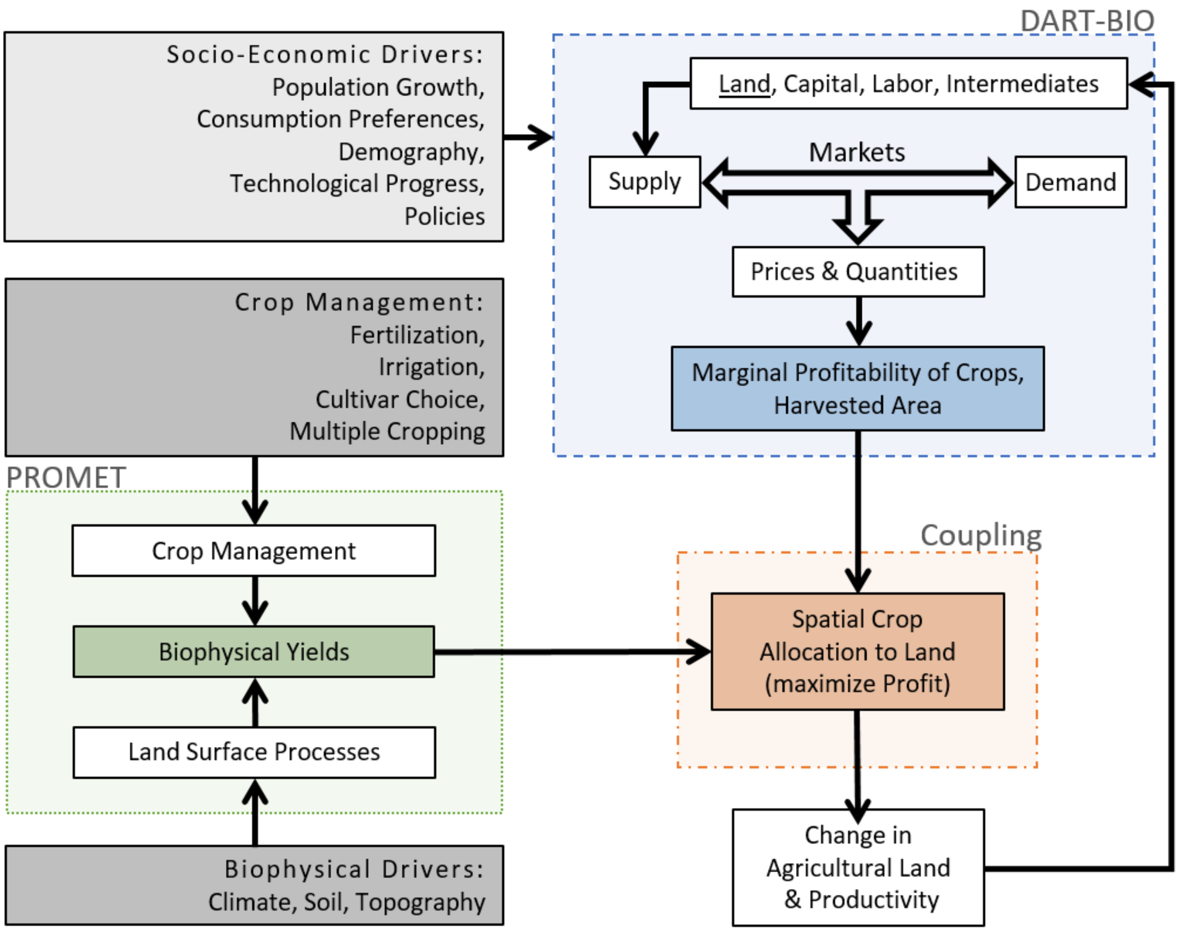

Figure B. Modified coupling approach of PROMET and DART-BIO to integratively assess the effect of land saving on agricultural markets and the resulting feedbacks on land saving.

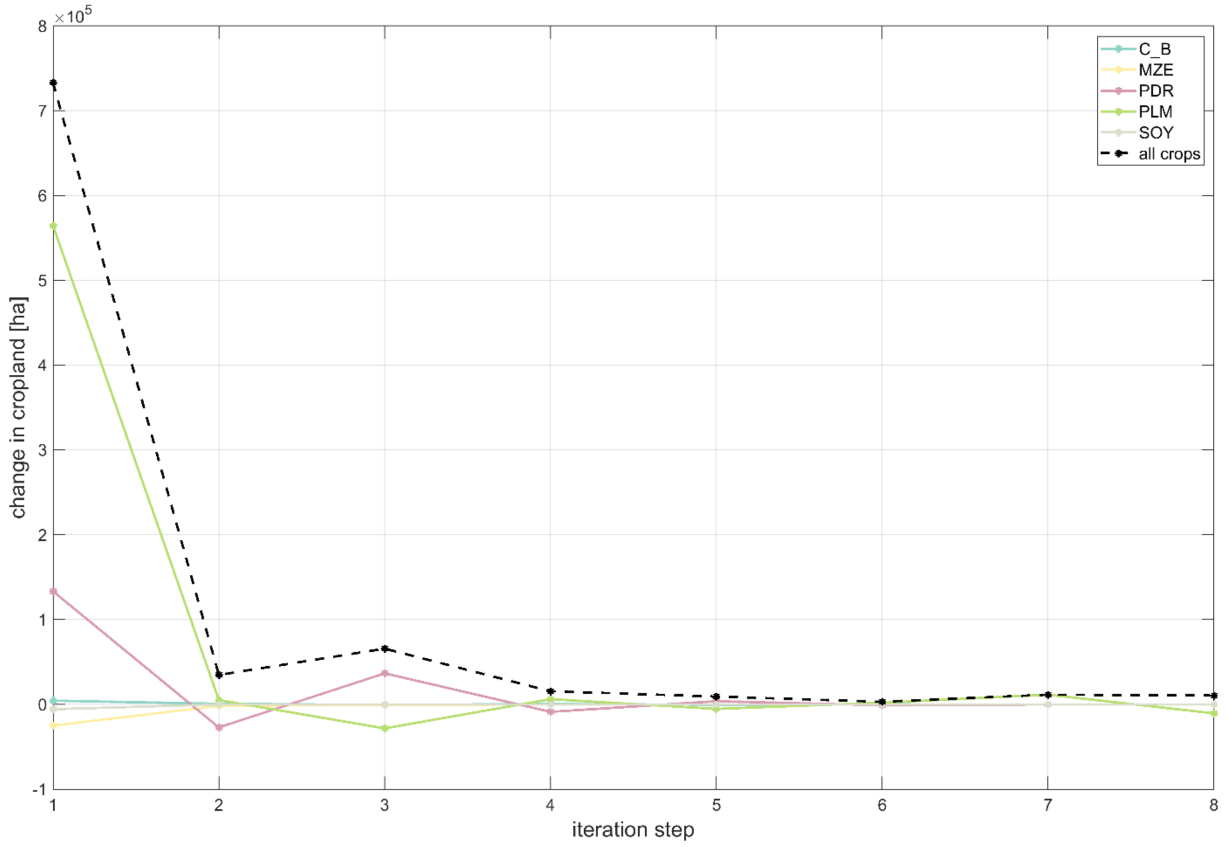

Figure C. Change in allocated cropland [hectare] per crop category and accumulated over all crops within the exemplary sub-region AEZ 6 in Malaysia and Indonesia. For each sub-region, a stable cropland allocation can be achieved after a different iteration step. However, iteration is carried out until a stable allocation is reached globally in all sub-regions.

References

1. Mauser W, Klepper G, Zabel F, Delzeit R, Hank T, Putzenlechner B, et al. Global biomass production potentials exceed expected future demand without the need for cropland expansion. Nat Commun. 2015;6. doi: 10.1038/ncomms9946.
