# Supplementary material for: Global cropland could be almost halved: Assessment of land saving potentials under different strategies and implications for agricultural markets
Source: PLoS One. 2022 Feb 22;17(2):e0263063. doi: 10.1371/journal.pone.0263063 (PMC8863228; doi:10.1371/journal.pone.0263063)
Supplement: S6 Appendix — (PDF) [file pone.0263063.s006.pdf]

S6 Appendix: Effect of different yield gap closing scenarios

We assessed the land saving potential for different yield gap closing scenarios from 50% to 100% yield gap closing. When yield gaps are closed by 50% instead of 80%, land saving potentials decrease for all land saving strategies by around -7 pp, resulting in global land saving potentials of 41% (BLS) to 29% (ULS). Assuming full yield gap closing (100%), on the other hand, increases the global land saving potential only by +3 pp to +4 pp compared to an 80% yield gap closing, so that globally, 51% (BLS) to 40% (ULS) of current cropland could be taken out of production. Since for all strategies the global land saving potential increases only by around 10 percentage points when yield gaps are fully closed instead of a 50% yield gap closing (Table A), our results suggest that the large potentials for land saving are not strictly tied to a highly-intensified agriculture, but persist also within lower yield gap closing scenarios.

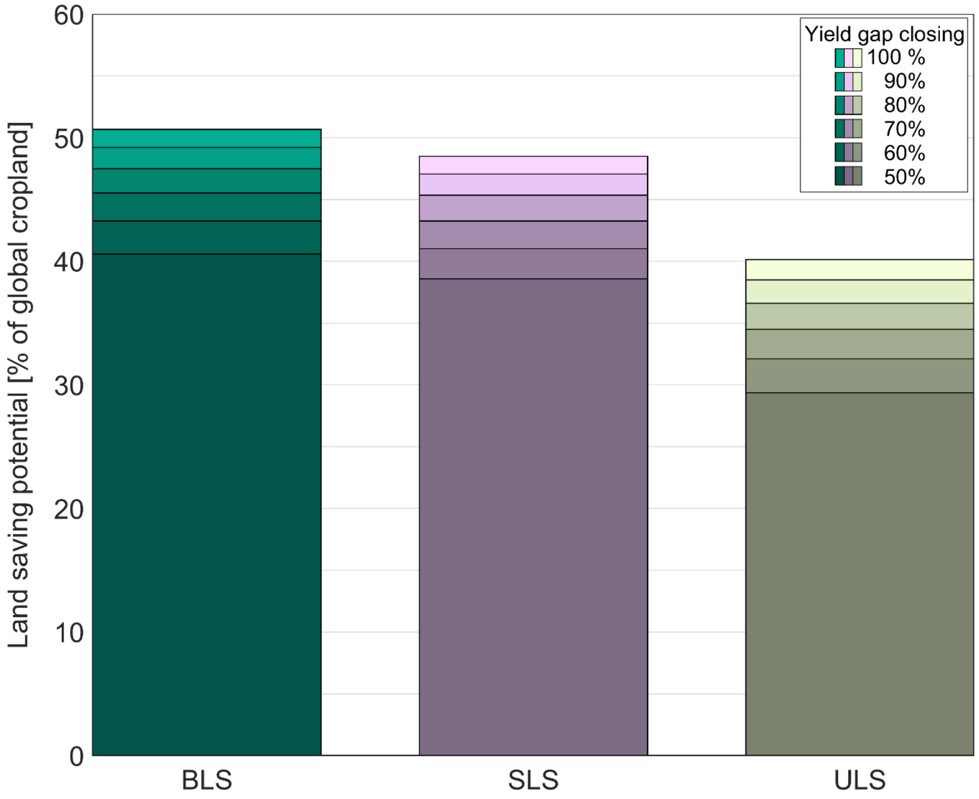

Figure. Land saving potentials for biophysical land saving (BLS), socio-economic land saving (SLS) and uniform land saving (ULS) for 50%, 60%, 70%, 80%, 90% and full (100%) yield gap closing. The land saving potentials displayed for the SLS are obtained with direct coupling without iteration.

Table A. Global land saving potentials [% of global cropland] for the three different land saving strategies biophysical land saving (BLS), socio-economic land saving (SLS) and uniform land saving (ULS) under different yield gap closing scenarios.

yield gap closing	BLS	SLS	ULS
50%	40.59	38.59	29.38
60%	43.27	41.03	32.11
70%	45.54	43.26	34.49
80%	47.50	45.36	36.60
90%	49.21	47.06	38.48
100%	50.69	48.51	40.15

Analyzing the sensitivity of global impacts on agricultural markets, our results show that the global average changes in production and prices due to the implementation of land saving are very robust against different yield gap closing scenarios. Looking at the BLS and the ULS strategies as an upper and lower boundary, we see that the changes between 50% and 100% yield gap closing scenarios are around 0.1% for crop production and between 0.4% and 0.6% for crop prices (Table B). This result is to be expected, as the correlation between yield gap closing and land saving is rather strong, so that there's less land available in the economic model by the degree by which we close yield gaps.

Table B. Global average changes of crop production and prices [%] compared to a baseline without land saving for the biophysical land saving (BLS) and the uniform land saving (ULS) strategy under different yield gap closing scenarios.

	Change in global crop production		Global average change of crop prices	
yield gap closing	BLS	ULS	BLS	ULS
50%	+2.87	+2.99	-8.53	-9.14
80%	+2.84	+2.90	-8.24	-8.73
100%	+2.84	+2.87	-8.12	-8.52
