# Supplementary material for: Global cropland could be almost halved: Assessment of land saving potentials under different strategies and implications for agricultural markets
Source: PLoS One. 2022 Feb 22;17(2):e0263063. doi: 10.1371/journal.pone.0263063 (PMC8863228; doi:10.1371/journal.pone.0263063)
Supplement: S7 Appendix — (PDF) [file pone.0263063.s007.pdf]

S7 Appendix: Supplementary results

Land saving potentials

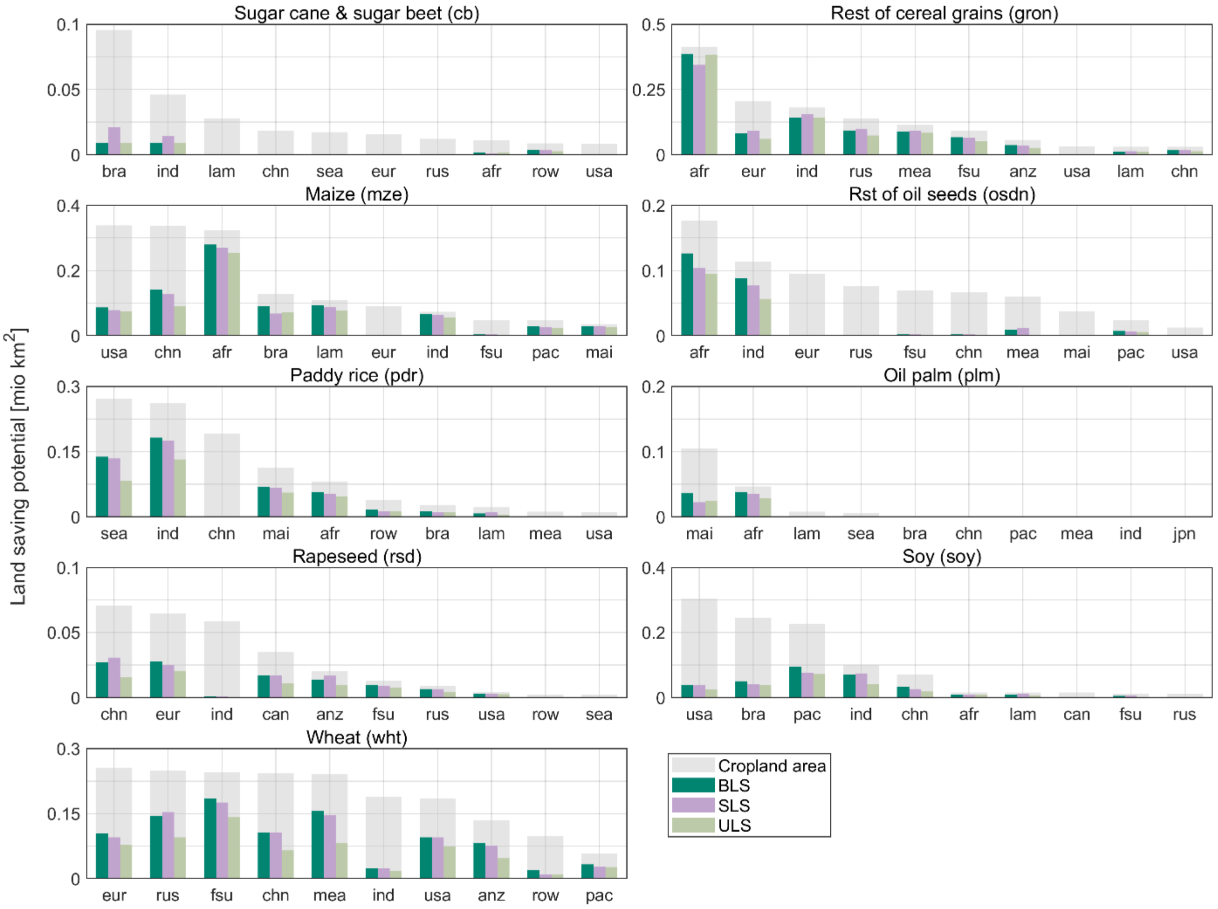

Figure A. Absolute land saving potential [million km^2] for the top 10 growing regions of each crop category. Grey bars show the current cropland extent, colored bars the area that could be saved under biophysical land saving (BLS), socio-economic land saving (SLS) and uniform land saving (ULS). Top growing regions are defined as the 10 regions with the largest statistical growing area of the crop category.

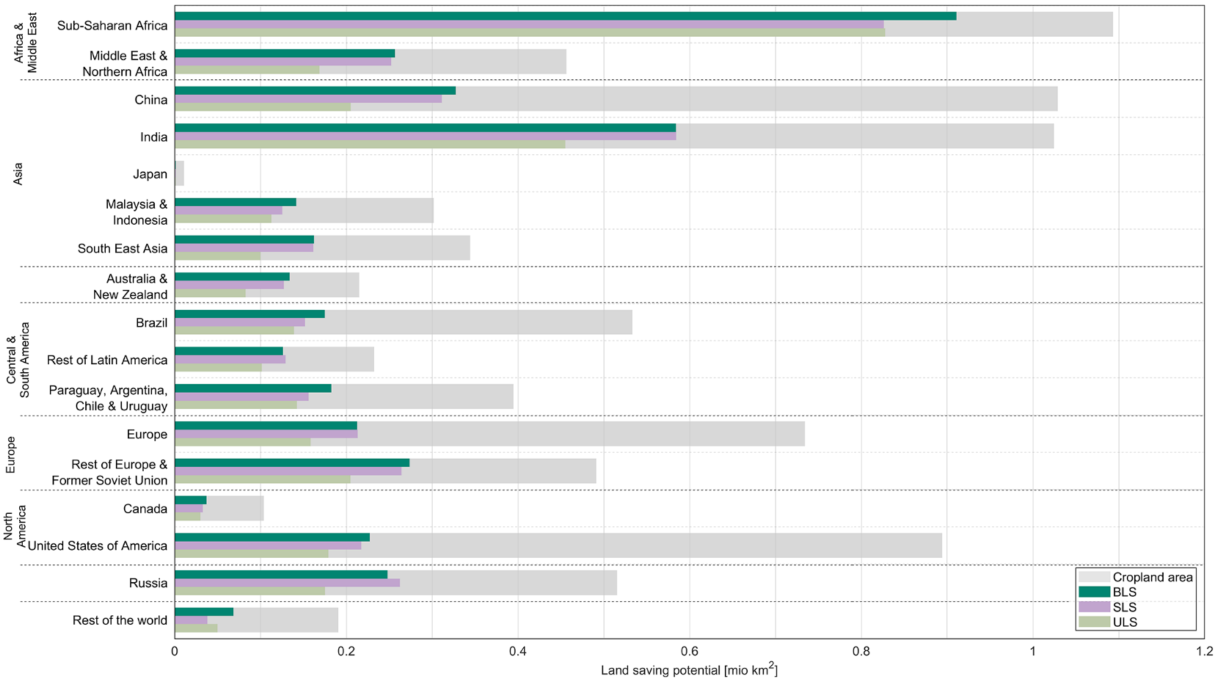

Figure B. Absolute land saving potential [million km^2] for the 17 study regions under biophysical land saving (BLS), socio-economic land saving (SLS) and uniform land saving (ULS). Grey bars show the current cropland extent, colored bars the area that could be saved under the three different land saving strategies within each region accumulated over all crops.

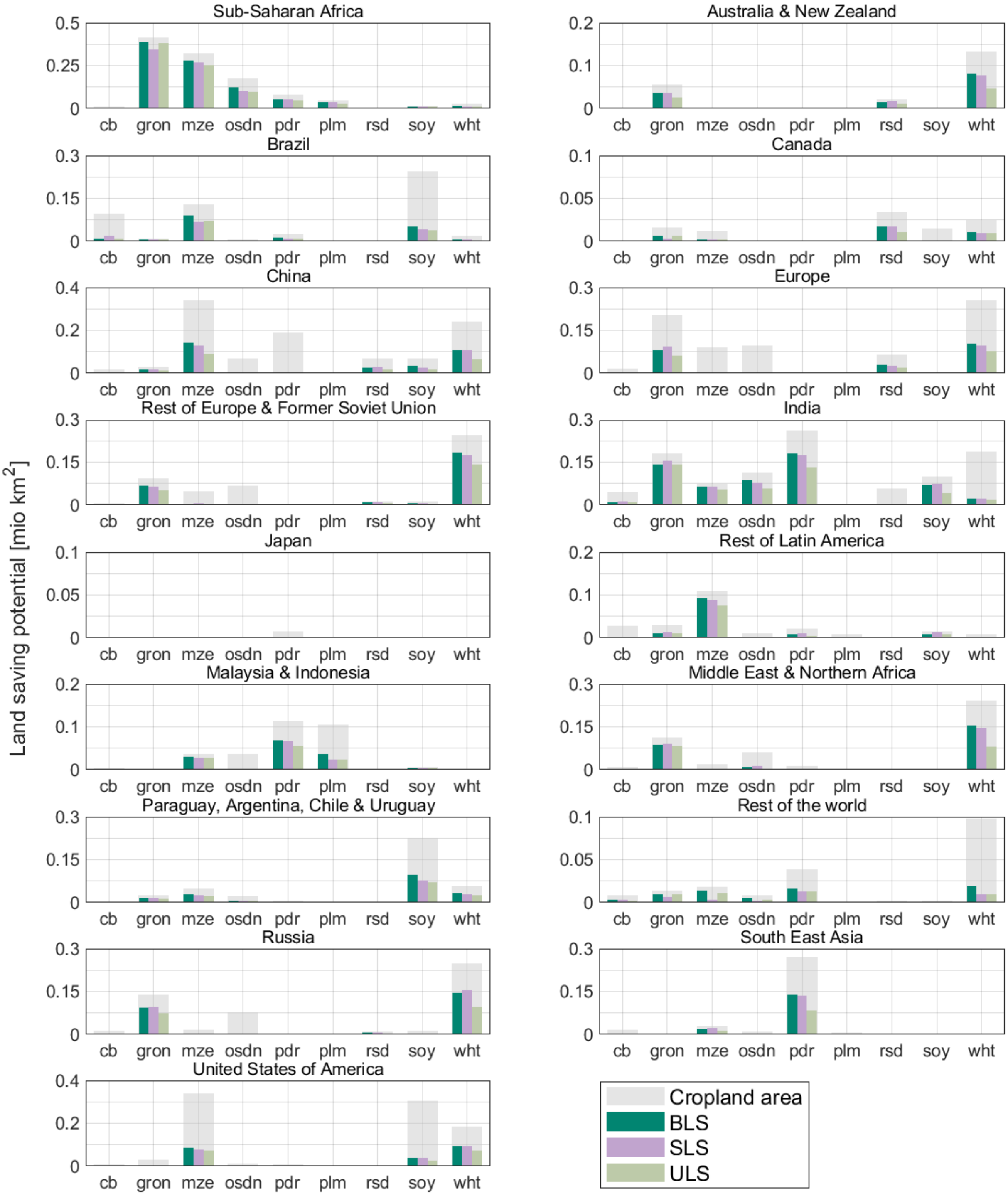

Figure C. Absolute land saving potential [million km^2] for each crop category in each of the 17 study regions under biophysical land saving (BLS), socio-economic land saving (SLS) and uniform land saving (ULS). Grey bars show the current cropland extent, colored bars the area that could be saved under the three different land saving strategies.

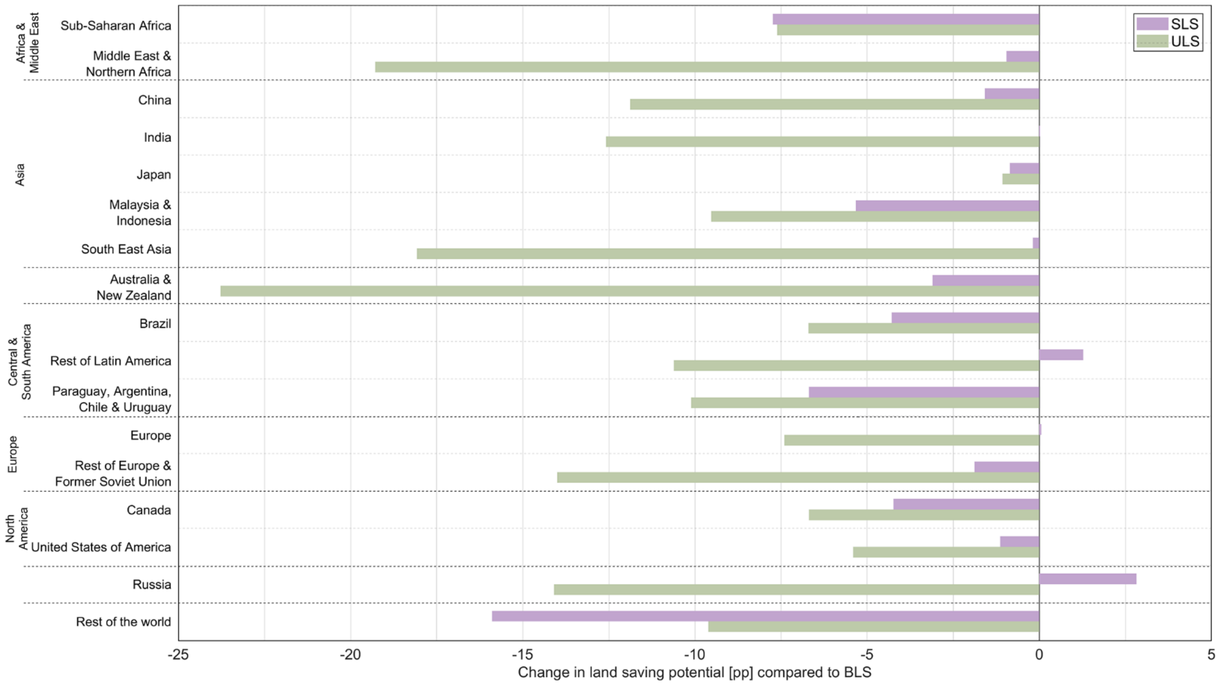

Figure D. Change in the regional land saving potential in percentage points [pp] compared to the biophysical land saving (BLS) as upper benchmark for realizable land saving.

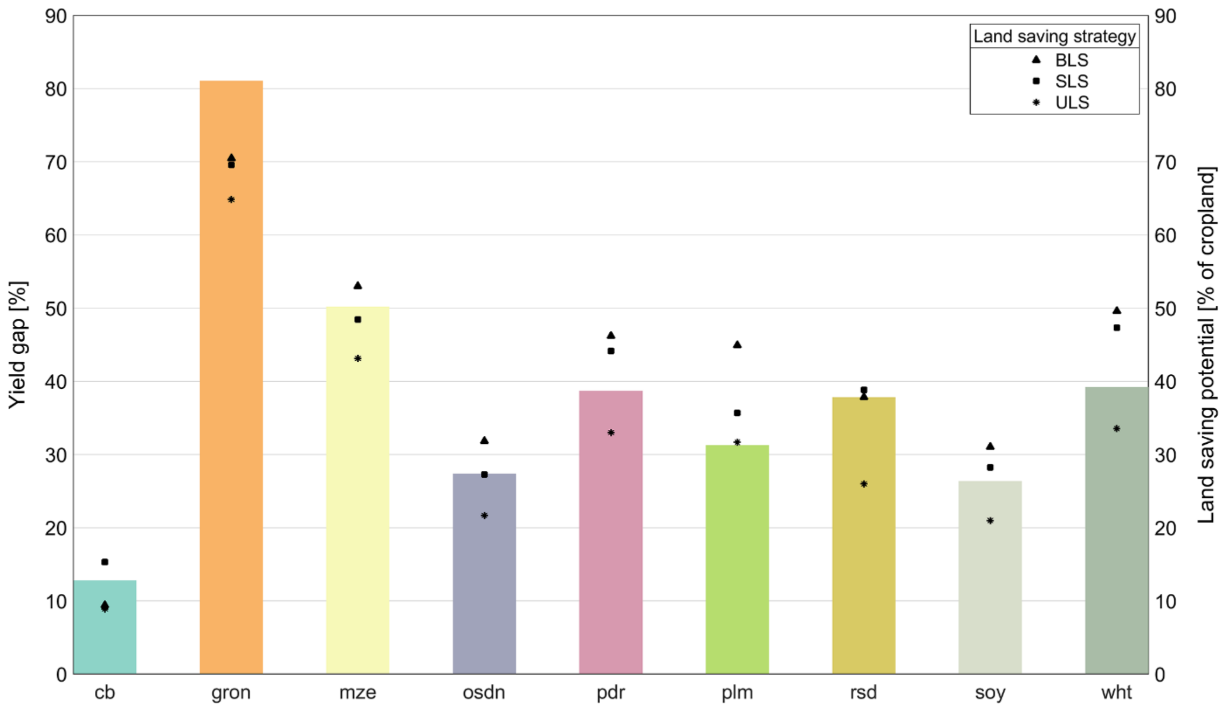

Figure E. Global area weighted yield gap [%] and land saving potential [% of global cropland] per crop category for the three land saving strategies biophysical land saving (BLS), socio-economic land saving (SLS) and uniform land saving (ULS). Yield gaps are displayed with bars and land saving potentials with markers. The yield gap is defined as the percentage difference between statistical and potential yields (1-statistical yield/ potential yield).

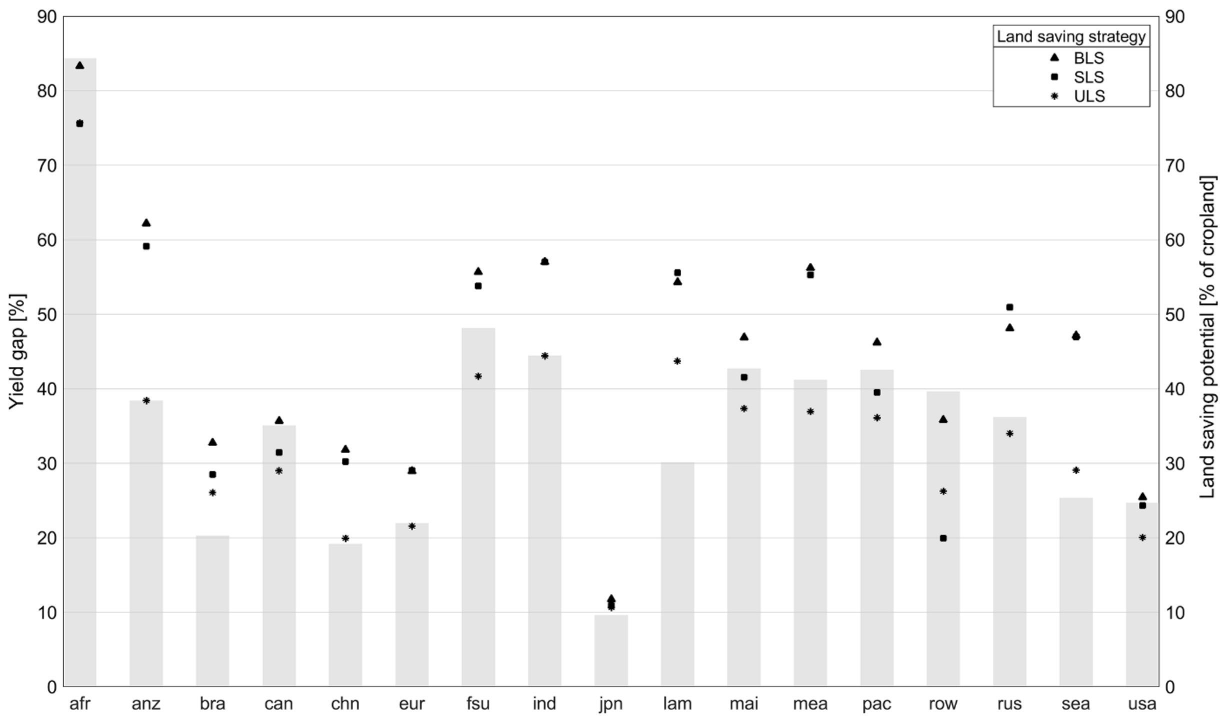

Figure F. Regional yield gap [%] area-weighted across all crop categories and potential for land saving with the three different land saving strategies. Yield gaps are displayed with bars and land saving potentials with markers. The yield gap is defined as the percentage difference between statistical and potential yields (1-statistical yield/ potential yield).

Table A. Relative land saving potentials [% of cropland] under the biophysical land saving (BLS) strategy.

region / crop category	cb	gron	mze	osdn	pdr	plm	rsd	soy	wht	overall
Sub-Saharan Africa	13.65	93.18	86.38	71.38	68.94	81.8	46.6	63.61	58.83	83.32
Australia & New Zealand	0	65.73	22.95	11.52	0	0	68.1	43.29	61.67	62.21
Brazil	9.25	49.84	70.2	5.26	49.28	0.96	22.86	20.6	32.21	32.77
Canada	0	41.77	21.04	23.7	0	0	48.72	0	43.02	35.67
China	0.92	54.37	41.91	3.36	0.12	0	38.52	46.69	43.85	31.81
Europe	0	39.41	0.15	0.1	0	0	42.92	8.51	40.67	28.97
Rest of Europe & Former Soviet Union	3.54	71.22	8.69	2.82	57.58	0	74.31	42.19	75.07	55.7
India	19.37	78.89	88.72	77.99	69.58	0	1.39	70.71	12.76	57.02
Japan	0	77.75	0	0	0	0	0	5.27	43.12	11.73
Rest of Latin America	0	33.23	85.86	27.18	36.68	10.29	0	61.14	14.58	54.31
Malaysia & Indonesia	42.92	0	81.93	0	61.18	34.27	0	82.53	0	46.88
Middle East & Northern Africa	11.43	76.74	10.89	15.88	0	0	45.99	0.04	64.69	56.24
Paraguay, Argentina, Chile & Uruguay	13.92	56.15	60.44	32.16	1.36	58.81	40.11	42.48	56.94	46.22
Rest of the world	42.35	73.14	73.2	62.8	42.75	0	0	0.02	19.87	35.84
Russia	0	67.5	24.07	0	17.14	0	73.29	0	57.64	48.11
South East Asia	0	56.6	72.45	0.14	51	0	0	39.1	23.32	47.14
United States of America	0.68	9.39	25.82	0	0	0	69.91	12.95	51.33	25.45
global	9.42	70.49	53.02	31.85	46.21	44.94	37.87	31.05	49.62	47.5

The potential is displayed for each region and each crop category, as well as per region accumulated over all crop categories (overall) and globally accumulated over all regions (global). For the different crop categories, the following abbreviations are used (for details see also Table in S2 Appendix): cb: sugar cane & sugar beet; gron: rest of cereal grains; mze: maize; osdn: rest of oil seeds; pdr: paddy rice; plm: oil palm; rsd: rapeseed; soy: soy; wht: wheat.

Table B. Relative land saving potentials [% of cropland] under the socio-economic land saving (SLS) strategy.

region / crop category	cb	gron	mze	osdn	pdr	plm	rsd	soy	wht	overall
Sub-Saharan Africa	9.62	83.36	83.43	58.77	65.31	76.96	33.85	57.11	36.59	75.59
Australia & New Zealand	0	63.98	-298.89	25.77	0	0	81.58	-18.04	57.11	59.11
Brazil	21.85	53.47	53.18	5.25	36.46	1.33	6.24	16.93	27.7	28.49
Canada	0	23.13	16.6	3.84	0	0	48.88	0	40	31.44
China	-0.68	55.82	38.04	3.11	0.28	0	43.49	37.57	43.6	30.24
Europe	0	45.09	0.05	0.32	0	0	38.64	8.37	37.35	29.03
Rest of Europe & Former Soviet Union	13.93	69.76	10.18	3.06	51.75	0	68.77	46.12	71.36	53.81
India	30.5	86.1	86.08	67.63	66.81	0	1.73	74.21	12.36	57.04
Japan	0	77.99	0	0	0	0	0	30.86	18.49	10.88
Rest of Latin America	0	42.47	80.99	24.26	48.07	11.76	0	78.4	19.88	55.58
Malaysia & Indonesia	53.61	0	80.8	0	58.77	21.6	0	79.06	0	41.56
Middle East & Northern Africa	-0.79	80.08	8.44	20.42	0	0	37.09	0.03	60.86	55.29
Paraguay, Argentina, Chile & Uruguay	14.73	57.14	56.73	29.37	1.49	55.9	46.85	34.12	47.62	39.53
Rest of the world	38.89	45.86	20.37	23.21	32.36	0	0	0.02	10.41	19.95
Russia	0	70.61	24.99	0	17.14	0	74.06	0	61.66	50.94
South East Asia	0	86.24	75.97	1.25	49.57	0	0	72.16	26.99	46.96
United States of America	7.62	8.94	23.09	0	0	0	68.52	12.32	51.64	24.31
global	15.31	69.6	48.45	27.25	44.15	35.69	38.83	28.22	47.34	45.01

The potential is displayed for each region and each crop category, as well as per region accumulated over all crop categories (overall) and globally accumulated over all regions (global). For the different crop categories, the following abbreviations are used (for details see also Table in S2 Appendix): cb: sugar cane & sugar beet; gron: rest of cereal grains; mze: maize; osdn: rest of oil seeds; pdr: paddy rice; plm: oil palm; rsd: rapeseed; soy: soy; wht: wheat. Within a SLS, negative land saving potentials can occur when less profitable crops are shifted to locations with less optimal growing conditions and accordingly lower yield potentials.

Table C. Relative land saving potentials [% of cropland] under the uniform land saving (ULS) strategy.

region / crop category	cb	gron	mze	osdn	pdr	plm	rsd	soy	wht	overall
Sub-Saharan Africa	13.65	92.85	78.28	53.56	57.94	61.53	30.24	52.79	42.75	75.71
Australia & New Zealand	0	46.16	6.88	8.19	0	0	47.51	23.25	34.99	38.43
Brazil	9.25	49.84	56.17	3.92	36.68	0.96	15.27	15.38	24.68	26.07
Canada	0	41.77	18.23	23.7	0	0	31.65	0	40.36	28.98
China	0.45	44.72	26.66	1.9	0.02	0	22.16	26.7	26.98	19.93
Europe	0	29.4	0.08	0.1	0	0	31.24	5.65	30.42	21.57
Rest of Europe & Former Soviet Union	3.54	55.74	0.65	1.08	45.74	0	61.09	7.87	57.61	41.7
India	19.37	78.89	75.14	49.91	50.31	0	0.97	40.79	9.53	44.44
Japan	0	77.75	0	0	0	0	0	0.24	38.28	10.67
Rest of Latin America	0	32.67	69.9	20.44	20.11	2	0	50.49	9.55	43.7
Malaysia & Indonesia	42.92	0	75.14	0	49.1	22.82	0	71.28	0	37.35
Middle East & Northern Africa	7.66	73.25	4.23	1.86	0	0	45.79	0.03	33.93	36.95
Paraguay, Argentina, Chile & Uruguay	13.92	49.46	46.85	25.49	1.13	45.87	21.26	32.13	46	36.11
Rest of the world	30.84	73.14	58.92	41.33	34.17	0	0	0.02	10.07	26.24
Russia	0	52.84	12.78	0	12.7	0	51.48	0	38.2	34.02
South East Asia	0	56.11	49.5	0.11	30.76	0	0	25.04	12.79	29.07
United States of America	0.68	8.15	22.21	0	0	0	62.84	8.2	39.96	20.04
global	8.93	64.86	43.14	21.68	33	31.68	25.98	21.01	33.54	36.6

The potential is displayed for each region and each crop category, as well as per region accumulated over all crop categories (overall) and globally accumulated over all regions (global). For the different crop categories, the following abbreviations are used (for details see also Table in S2 Appendix): cb: sugar cane & sugar beet; gron: rest of cereal grains; mze: maize; osdn: rest of oil seeds; pdr: paddy rice; plm: oil palm; rsd: rapeseed; soy: soy; wht: wheat.

Impacts on agricultural markets

Table D. Relative changes [%] in crop production compared to statistical reference production under biophysical land saving (BLS) for each crop category within each region and accumulated over all crop categories (overall).

region / crop category	cb	gron	mze	osdn	pdr	plm	rsd	soy	wht	overall
Sub-Saharan Africa	-3.96	9.95	7.93	5.1	6.6	3.29	-7.68	1.29	-22.83	1.49
Australia & New Zealand	-6.41	-9.34	-5.45	-3.46	-3.64	0	28.36	3.26	10.07	1.5
Brazil	23.31	5.38	6.54	-1.21	2.31	-34.34	-1.63	-16.01	-5.34	2.39
Canada	-1.26	-6.21	-12.6	-6.16	0	0	-14.31	-25.77	-17.35	-7.49
China	-0.19	24.15	10.59	1.09	-2.88	-21.17	29.71	107.87	16.79	4.13
Europe	-2.51	1.4	-11.76	-12.5	-7.55	0	1.2	-32.12	-3.3	-2.09
Rest of Europe & Former Soviet Union	-0.35	21.64	-1.44	-9.35	-0.99	0	62.23	-8.83	12.06	2.92
India	5.91	23.07	28.58	30.31	8.65	0	8.17	22.35	3.49	4.37
Japan	-5.58	-20.31	0	0	-0.17	0	0	-41.17	-32.1	-1.88
Rest of Latin America	-0.46	11.77	64.5	3.99	6.46	-4.48	0	37.03	-32.88	4.02
Malaysia & Indonesia	55.99	0	82.49	6.05	13.19	22.15	0	940.92	0	14.25
Middle East & Northern Africa	-1.41	-3.86	-39.75	-4.46	-0.4	0	-9.72	-59.88	-10.16	-4.62
Paraguay, Argentina, Chile & Uruguay	1.91	11.2	8.79	31.36	-2.51	13.03	6.12	16.18	15.58	10.48
Rest of the world	8.17	7.82	-27.07	12.77	2.56	-18.14	-26.07	-58.76	41.7	3.37
Russia	-3.81	26.93	3.52	-8.38	0.52	0	20.5	-60.07	32.63	5.71
South East Asia	-1.97	23.6	51.27	-31.67	14.59	-12.99	-54.63	192.74	-35.03	6.02
United States of America	1.37	0.82	5.94	-2.55	-5.25	0	44.27	-5.48	19.45	6.15

For the different crop categories, the following abbreviations are used (for details see also Table in S2 Appendix): cb: sugar cane & sugar beet; gron: rest of cereal grains; mze: maize; osdn: rest of oil seeds; pdr: paddy rice; plm: oil palm; rsd: rapeseed; soy: soy; wht: wheat.

Table E. Relative changes [%] in crop production compared to statistical reference production under socio-economic land saving (SLS) for each crop category within each region and accumulated over all crop categories (overall).

region / crop category	cb	gron	mze	osdn	pdr	plm	rsd	soy	wht	overall
Sub-Saharan Africa	-3.6	8.34	7.92	4.06	6.55	3.55	-8.87	0.99	-30.9	1.46
Australia & New Zealand	-7.2	-9.97	-6.11	2.1	-3.59	0	41.57	4.83	12.9	2.95
Brazil	22.73	5.45	5.72	-1.15	2.35	-35.56	-1.71	-16.01	-2.67	2.27
Canada	-1.21	-6.98	-12.12	-4.75	0	0	-14.27	-26.28	-16.25	-7.32
China	0.05	25.06	10.65	1.78	-2.8	-21.46	28.35	100.15	17.15	4.22
Europe	-2.43	1.71	-11.12	-12.34	-7.32	0	0.01	-31.78	-3.29	-2.2
Rest of Europe & Former Soviet Union	-0.33	21.37	-0.86	-9.35	-0.97	0	54.99	-7.96	11.39	2.75
India	5.8	27.18	25.68	26.28	8.51	0	7.69	24.8	3.43	4.17
Japan	-5.39	-23.19	0	0	-0.15	0	0	-39.71	-34.28	-2
Rest of Latin America	-0.53	20.02	55.7	0.3	6.58	-4.74	0	75.17	-28.89	3.54
Malaysia & Indonesia	56.28	0	81.75	8.43	13.23	23.08	0	968.44	0	14.77
Middle East & Northern Africa	-1.08	-1.33	-38.53	-3.13	-0.3	0	-10.3	-59.66	-10.85	-4.5
Paraguay, Argentina, Chile & Uruguay	2.41	10.39	11.78	33.4	-2.32	12.16	9.25	15.61	14.6	11.07
Rest of the world	6.73	-19.51	-33.25	14.4	1.74	-18.56	-21.57	-50.43	30.63	2.18
Russia	-3.73	27.67	2.92	-8.92	0.52	0	20.84	-62.47	36.53	6.13
South East Asia	-1.88	37.06	51.9	-35.67	14.7	-13.31	-62.77	612.07	-38	6.81
United States of America	1.5	0.04	6.4	-1.83	-4.63	0	43.62	-6.19	21.43	6.38

For the different crop categories, the following abbreviations are used (for details see also Table in S2 Appendix): cb: sugar cane & sugar beet; gron: rest of cereal grains; mze: maize; osdn: rest of oil seeds; pdr: paddy rice; plm: oil palm; rsd: rapeseed; soy: soy; wht: wheat.

Table F. Relative changes [%] in crop production compared to statistical reference production under uniform land saving (ULS) for each crop category within each region and accumulated over all crop categories (overall).

region / crop category	cb	gron	mze	osdn	pdr	plm	rsd	soy	wht	overall
Sub-Saharan Africa	-3.82	10.08	7.19	3.74	6.3	2.46	-12.99	1.02	-25.45	1.42
Australia & New Zealand	-5.66	-10.53	-3.3	1.88	-3.4	0	24.47	6.36	9.24	2.01
Brazil	23.45	5.67	5.84	-1.1	2.3	-35.73	-1.59	-15.1	-1.44	2.56
Canada	-1.24	-6.47	-11.01	-5.74	0	0	-14.44	-24.36	-15.47	-7.29
China	0.25	23.83	10.65	3.11	-2.43	-21.06	28.68	94.6	15.92	4.49
Europe	-2.51	0.91	-9.9	-11.95	-7.58	0	0.96	-28.69	-2.74	-2.23
Rest of Europe & Former Soviet Union	-0.11	17.73	0.85	-7.62	-0.95	0	50.51	-16.1	10.17	2.81
India	6.23	24.44	22.64	21.39	8.61	0	7.86	17.31	3.67	4.1
Japan	-5.95	-20.35	0	0	-0.22	0	0	-39.8	-30.26	-2.38
Rest of Latin America	-0.29	14.14	47.48	3.38	6.5	-4.48	0	27.2	-30.21	2.7
Malaysia & Indonesia	57.22	0	77.42	9.45	13.19	23.04	0	710.43	0	14.12
Middle East & Northern Africa	-1.49	-2.74	-35.44	-4.78	-0.29	0	-5	-54.8	-11.01	-4.42
Paraguay, Argentina, Chile & Uruguay	2.32	10.74	9.5	32.29	-2.26	12.81	3.57	17.87	15.59	11.65
Rest of the world	8.39	8.79	-24.66	12.22	2.62	-18.49	-22.52	-55.83	41.94	3.55
Russia	-3.25	22.99	4.99	-6.33	0.68	0	15.14	-50.82	28.86	5.7
South East Asia	-0.92	26.27	31.54	-19.5	15.64	-12.49	-48.69	171.74	-30.4	6.68
United States of America	1.42	0.47	6.95	-1.12	-5.23	0	47.06	-3.72	18.94	6.62

For the different crop categories, the following abbreviations are used (for details see also Table in S2 Appendix): cb: sugar cane & sugar beet; gron: rest of cereal grains; mze: maize; osdn: rest of oil seeds; pdr: paddy rice; plm: oil palm; rsd: rapeseed; soy: soy; wht: wheat.

Table G. Relative changes [%] in crop prices under biophysical land saving (BLS) for each crop category within each region and accumulated over all crop categories (overall).

region / crop category	cb	gron	mze	osdn	pdr	plm	rsd	soy	wht	overall
Sub-Saharan Africa	-3.22	-17.66	-15.96	-13.41	-13.98	-12.51	-9.07	-12.32	-12.52	-6.13
Australia & New Zealand	-3.64	-7.3	-5.71	-9.9	-1.7	-22.12	-20.33	-13.73	-16.48	-6.73
Brazil	-11.71	-13.69	-14.17	-9.11	-12.5	-14.35	-9.51	-3.94	-13.99	-8.77
Canada	-5.28	-7.27	-10.01	-10.61	-7.45	-18.78	-7.79	-7.73	-3.96	-7.62
China	-1.88	-24.8	-23.28	-8.78	-4.22	-17.51	-20.96	-14.97	-20.2	-7.69
Europe	-2.02	-11.32	-4.64	-3.93	-4.35	0	-11.54	-7	-10.95	-4.95
Rest of Europe & Former Soviet Union	-3.95	-18.41	-6.82	-5.5	-2.9	-19.87	-20.76	-12.73	-17.8	-7.06
India	-13.6	-29.31	-34.33	-36.78	-37.32	-17.36	-7.76	-24.47	-0.41	-9.77
Japan	-2.93	-12.51	-15.8	-10.44	-0.7	-17.13	-10.31	-12.26	-13.21	-1.45
Rest of Latin America	-2.17	-15.1	-22.64	-11.69	-17.35	-11.02	-10.42	-14.49	-11.47	-7.41
Malaysia & Indonesia	-19.94	-13.4	-37.73	7.27	-48.66	-35.63	-10.6	-27.35	-13.6	-23.03
Middle East & Northern Africa	-5.51	-10.78	-10.61	-7.87	-1.91	-18.33	-9.86	-13.1	-11.32	-3.95
Paraguay, Argentina, Chile & Uruguay	-7.52	-17.32	-17.21	-15.71	-3	-19.08	-14.38	-15.83	-20.06	-13.29
Rest of the world	-15.58	-13.29	-15.44	-13.52	-6.87	-24.92	-6.14	-12.62	-16.95	-6.57
Russia	-3.21	-21.88	-10.15	-2.23	-7.63	-19.11	-20.94	-10.24	-25.86	-7.91
South East Asia	-3.88	-27.39	-37.31	-7.81	-25.81	-14.81	-6.51	-13.95	-12.69	-13.58
United States of America	-8.9	-12.53	-12.92	-9.4	-6.78	-15.87	-12.85	-13.95	-20.34	-10.39

For the different crop categories, the following abbreviations are used (for details see also Table in S2 Appendix): cb: sugar cane & sugar beet; gron: rest of cereal grains; mze: maize; osdn: rest of oil seeds; pdr: paddy rice; plm: oil palm; rsd: rapeseed; soy: soy; wht: wheat.

Table H. Relative changes [%] in crop prices under socio-economic land saving (SLS) for each crop category within each region and accumulated over all crop categories (overall).

region / crop category	cb	gron	mze	osdn	pdr	plm	rsd	soy	wht	overall
Sub-Saharan Africa	-3.56	-15.58	-15.81	-12.28	-13.87	-13.09	-9.32	-12.07	-12.25	-6.18
Australia & New Zealand	-4.45	-7.46	-5.75	-13.01	-1.99	-22.68	-24.67	-15.44	-17.1	-7.43
Brazil	-11.47	-13.88	-13.27	-9.25	-12.82	-14.29	-9.11	-4.17	-13.9	-8.79
Canada	-5.38	-7.05	-9.77	-10.51	-7.09	-19.24	-7.95	-7.84	-4.39	-7.71
China	-2	-25.47	-23.31	-9.53	-4.42	-17.82	-20.46	-15	-20.5	-7.99
Europe	-2.05	-11.63	-4.81	-4.08	-4.32	0	-10.93	-7.44	-10.8	-5.01
Rest of Europe & Former Soviet Union	-3.93	-18.46	-7.08	-5.66	-2.89	-20.36	-19.45	-13.33	-17.38	-7
India	-13.38	-32.52	-32.56	-33.11	-36.95	-17.84	-7.81	-27.69	-0.31	-9.64
Japan	-2.9	-12.92	-15.37	-10.43	-0.75	-17.55	-10.51	-12.48	-13.14	-1.56
Rest of Latin America	-1.99	-17.38	-21.05	-10.18	-17.47	-10.97	-10.55	-15.75	-11.59	-7.15
Malaysia & Indonesia	-19.89	-12.68	-37.01	7.81	-48.7	-36.57	-10.61	-28.35	-13.5	-23.34
Middle East & Northern Africa	-5.73	-11.04	-10.39	-8.07	-1.99	-18.76	-9.87	-13.29	-11.24	-4.07
Paraguay, Argentina, Chile & Uruguay	-7.86	-17.79	-17.86	-16.49	-3.63	-19.06	-16.1	-15.88	-19.55	-13.63
Rest of the world	-13.95	-10.98	-14.95	-13.45	-5.7	-25.57	-6.95	-12.83	-15.57	-5.99
Russia	-3.07	-22.34	-9.88	-1.71	-7.54	-19.41	-20.89	-10.3	-27.01	-8.15
South East Asia	-3.83	-38.69	-37.23	-5.44	-25.82	-15.17	-6.13	-16.99	-12.42	-13.87
United States of America	-8.87	-12.43	-13.02	-9.57	-6.82	-16.18	-12.98	-13.87	-20.86	-10.49

For the different crop categories, the following abbreviations are used (for details see also Table in S2 Appendix): cb: sugar cane & sugar beet; gron: rest of cereal grains; mze: maize; osdn: rest of oil seeds; pdr: paddy rice; plm: oil palm; rsd: rapeseed; soy: soy; wht: wheat.

Table I. Relative changes [%] in crop prices under uniform land saving (ULS) strategy for each crop category within each region and accumulated over all crop categories (overall).

region / crop category	cb	gron	mze	osdn	pdr	plm	rsd	soy	wht	overall
Sub-Saharan Africa	-3.72	-17.87	-14.93	-12.16	-13.64	-11.22	-8.49	-11.73	-12.08	-6.32
Australia & New Zealand	-5.04	-6.9	-6.81	-12.28	-2.77	-22.87	-19.4	-14.54	-15.86	-7.72
Brazil	-11.84	-13.96	-13.46	-9.33	-12.71	-14.49	-9.11	-4.57	-13.97	-9.02
Canada	-5.72	-7.46	-9.92	-10.95	-7.95	-19.26	-7.85	-8.09	-4.91	-7.98
China	-2.38	-24.43	-22.99	-11.48	-4.79	-17.93	-20.21	-14.69	-19.71	-8.63
Europe	-2.32	-11.17	-5.47	-4.57	-4.69	0	-10.95	-7.69	-10.75	-5.34
Rest of Europe & Former Soviet Union	-4.47	-16.97	-8.02	-6.89	-3.07	-20.19	-18.69	-11.07	-16.35	-7.4
India	-14.27	-30.61	-30.29	-29.67	-36.86	-17.42	-10.71	-21.61	-1.54	-10.06
Japan	-3.24	-12.66	-15.14	-10.8	-0.93	-17.14	-10.26	-12.25	-12.88	-1.87
Rest of Latin America	-2.78	-15.82	-19.7	-11.68	-17.88	-11.72	-10.4	-14.2	-11.29	-7.36
Malaysia & Indonesia	-20.47	-13.78	-34.1	7.62	-49.03	-36.82	-10.88	-21.48	-13.21	-23.36
Middle East & Northern Africa	-5.92	-10.91	-10.37	-7.95	-2.5	-18.36	-10.01	-13.12	-10.76	-4.43
Paraguay, Argentina, Chile & Uruguay	-8.19	-17.42	-16.95	-16.08	-3.99	-19.2	-12.41	-16.19	-19.84	-13.75
Rest of the world	-16.14	-13.62	-14.75	-12.84	-7.51	-25.7	-6.58	-12.6	-16.7	-7.15
Russia	-3.63	-20.36	-11.03	-4.7	-8.2	-19.41	-18.18	-10.83	-24.12	-8.09
South East Asia	-4.82	-29.36	-30.45	-7.8	-27.38	-15.72	-7.14	-13.81	-12.4	-14.69
United States of America	-9.47	-12.73	-13.36	-9.78	-7.22	-16.36	-13.01	-14.7	-19.93	-10.79

For the different crop categories, the following abbreviations are used (for details see also Table in S2 Appendix): cb: sugar cane & sugar beet; gron: rest of cereal grains; mze: maize; osdn: rest of oil seeds; pdr: paddy rice; plm: oil palm; rsd: rapeseed; soy: soy; wht: wheat.

Land saving potential: Global maps

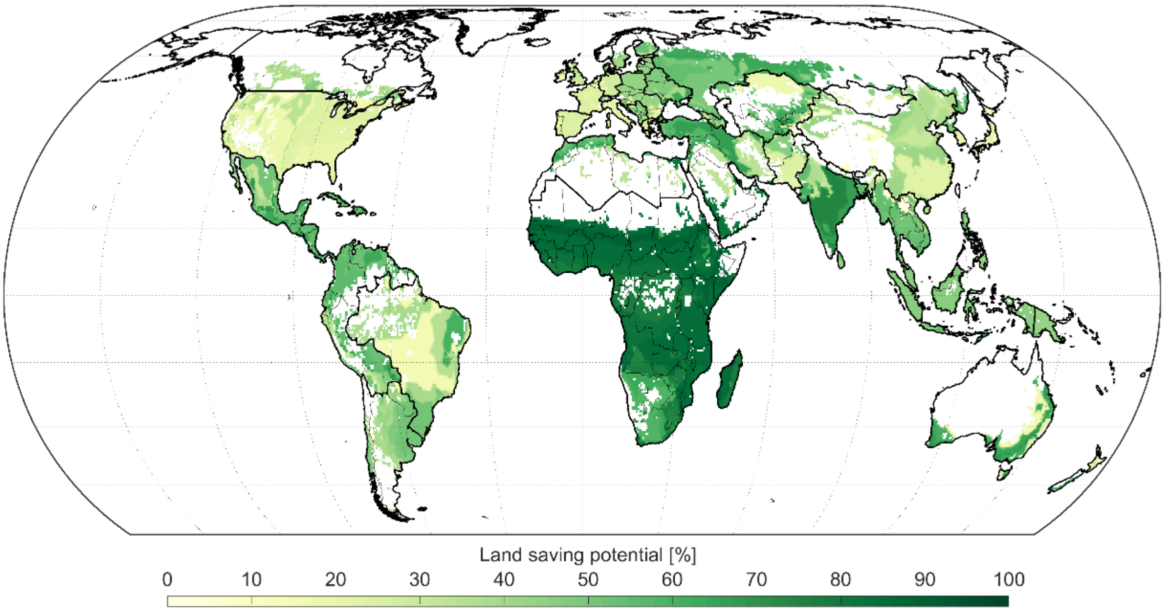

Figure G. Global map of the biophysical land saving potential (BLS). The potential describes the percentage share of total cropland (summed up over all considered crops according to Monfreda et al.[1], see S2 Appendix) that can be taken out of production within the sub-region. As land saving can only be implemented on current cropland areas, land that is currently not used as cropland is masked out. The thick region lines show the aggregated 17 study regions (see S3 Appendix), while the country boarders are displayed according to the global administrative areas of GADM version 2.8. Reprinted from GADM (https://gadm.org/) under a CC BY license, with permission from GADM, original copyright 2012.

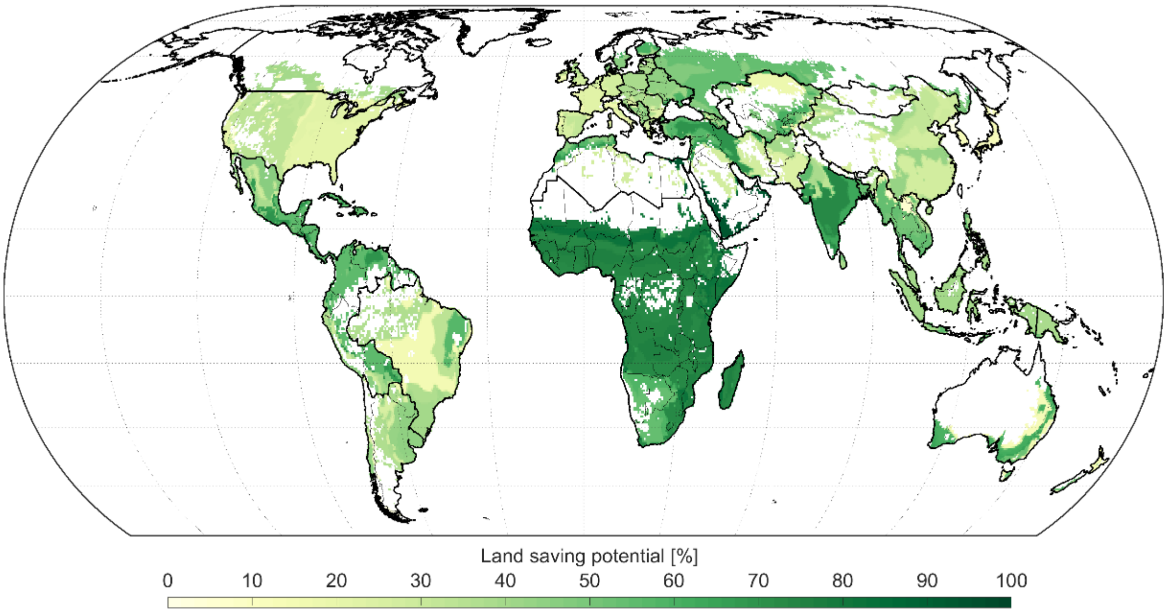

Figure H. Global map of the socio-economic land saving potential (SLS). The potential describes the percentage share of total cropland (summed up over all considered crops according to Monfreda et al.[1], see S2 Appendix) that can be taken out of production within the sub-region. As land saving can only be implemented on current cropland areas, land that

is currently not used as cropland is masked out. The thick region lines show the aggregated 17 study regions (see S3 Appendix), while the country boarders are displayed according to the global administrative areas of GADM version 2.8. Reprinted from GADM (https://gadm.org/) under a CC BY license, with permission from GADM, original copyright 2012.

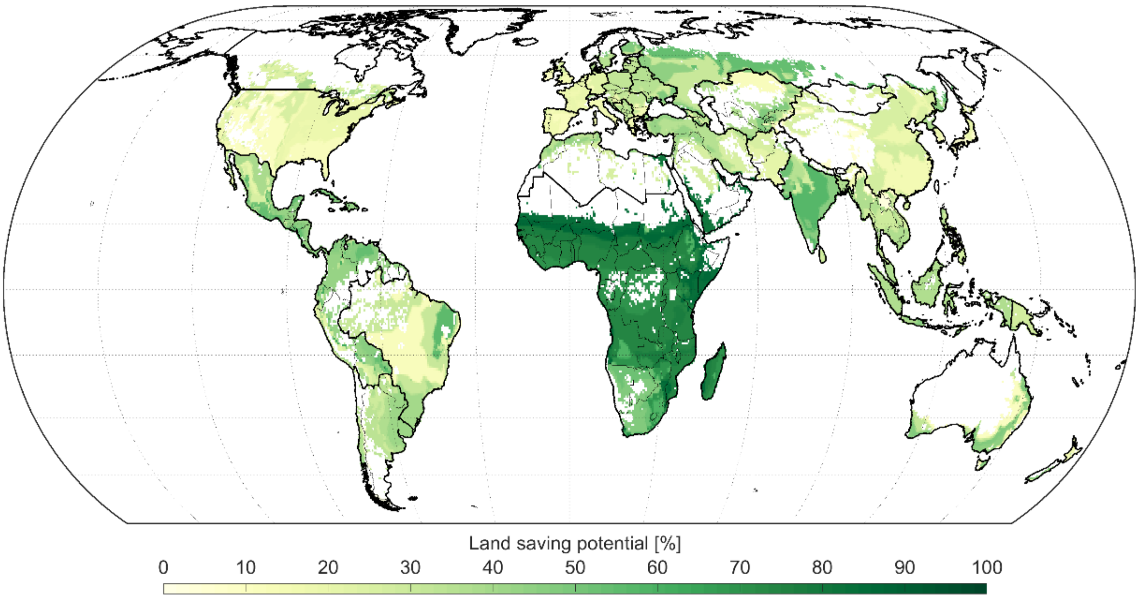

Figure I. Global map of the uniform land saving potential (ULS). The potential describes the percentage share of total cropland (summed up over all considered crops according to Monfreda et al.[1], see S2 Appendix) that can be taken out of production within the sub-region. As land saving can only be implemented on current cropland areas, land that is currently not used as cropland is masked out. The thick region lines show the aggregated 17 study regions (see S3 Appendix), while the country boarders are displayed according to the global administrative areas of GADM version 2.8. Reprinted from GADM (https://gadm.org/) under a CC BY license, with permission from GADM, original copyright 2012.

1. Monfreda C, Ramankutty N, Foley JA. Farming the planet: 2. Geographic distribution of crop areas, yields, physiological types, and net primary production in the year 2000. Global Biogeochemical Cycles. 2008;22(1). doi: 10.1029/2007GB002947.
