# Supplementary material for: Global cropland could be almost halved: Assessment of land saving potentials under different strategies and implications for agricultural markets
Source: PLoS One. 2022 Feb 22;17(2):e0263063. doi: 10.1371/journal.pone.0263063 (PMC8863228; doi:10.1371/journal.pone.0263063)
Supplement: S8 Appendix — (PDF) [file pone.0263063.s008.pdf]

S8 Appendix: Carbon sequestration potential of land saving

Saved land that has been taken out of agricultural use could potentially be used to sequester carbon by the recovery of natural vegetation. Studies have shown that e.g. forest recovery can play an important role for climate change mitigation [1]. To roughly estimate the sequestration potential by the recovery of natural vegetation on saved land, we assume that the potential natural vegetation type reestablishes as secondary vegetation, which has a reduced capacity for carbon storage in comparison to the original primary vegetation. Thus, the results of this calculation refer to the additionally saved carbon in the vegetation and soil layer after the time it takes to transform cropland to fully recovered potential secondary vegetation.

Therefore, we apply the spatially explicit bookkeeping model of land use emissions BLUE [2], in which the average carbon storage of 11 different primary and secondary vegetation types including their soils as well as the spatial distribution of the potential natural vegetation are deposited.

To obtain the additional carbon storage by the recovery of natural vegetation, the carbon sequestration of the potential secondary vegetation (including soils) is subtracted with the previous carbon sequestered by croplands (including soils), that are also deposited in the BLUE model. Current cropland areas used for the carbon sequestration calculation refer to the spatial distribution of harvested area according to Monfreda et al. [3] for the considered crops (see S2 Appendix) that are scaled to the area used in the GTAP database 9 [4] to be consistent with the economic model. Harvested areas are converted to (physical) growing area in order to avoid double counting of an area in case of more than one harvest per year by using a multiple cropping factor (harvested area/growing area) derived from the MIRCA dataset [5].

The calculation of the carbon balance takes place at 0.5° degree spatial resolution and is based on the sub-regional land saving potential of all crops across the sub-region.

We find that a recovery of natural vegetation on saved land of the investigated crops could globally sequester between 31 to 41 Gt more carbon than current cropland on the same area, which is equivalent to 114 Gt to 151 Gt CO_2. The considered crops in this study in total account for around 9 million km^2 globally. Table A shows the result of the carbon saving by recovery of the natural vegetation on saved land according to the different strategies for the considered crops, while Table B relates the results to the entire global cropland area by assuming that the land saving potential results of the considered crops can be transferred in general to all crops and the total global cropland, that globally accounts for approx. 15 million km^2.

Table A. Carbon sequestration potential of land saving by the recovery of natural vegetation in Gt C and Gt CO_2 and the corresponding globally recovered area in km^2 for the three different saving strategies for the considered crops (see S2 Appendix).

	Area [million km^2]	Additional carbon sequestration potential by land saving [Gt C]	Additional carbon sequestration potential by land saving [Gt CO_2]
BLS	4.0	41	151
SLS	3.8	39	144
ULS	3.1	31	114

Table B. Carbon sequestration potential of land saving by the recovery of natural vegetation in Gt C and Gt CO_2 and the corresponding globally recovered area in km^2 for the three different saving strategies, assuming that the considered crops are representative for the entire global cropland area.

	Area [million km^2]	Additional carbon sequestration potential by land saving [Gt C]	Additional carbon sequestration potential by land saving [Gt CO_2]
BLS	6.3	66	242
SLS	3.1	63	231
ULS	4.9	50	185

References

1. Poorter L, Craven D, Jakovac Catarina C, van der Sande Masha T, Amissah L, Bongers F, et al. Multidimensional tropical forest recovery. Science. 2021;374(6573):1370-6. doi: 10.1126/science.abh3629.
2. Hansis E, Davis SJ, Pongratz J. Relevance of methodological choices for accounting of land use change carbon fluxes. Global Biogeochemical Cycles. 2015;29(8):1230-46. doi: 10.1002/2014GB004997.

3. Monfreda C, Ramankutty N, Foley JA. Farming the planet: 2. Geographic distribution of crop areas, yields, physiological types, and net primary production in the year 2000. Global Biogeochemical Cycles. 2008;22(1):GB1022. doi: 10.1029/2007GB002947.
4. Aguiar A, Narayanan B, McDougall R. An Overview of the GTAP 9 Data Base. Journal of Global Economic Analysis. 2016;1(1):181-208. doi: 10.21642/jgea.010103af.
5. Portmann FT, Siebert S, Döll P. MIRCA2000—Global monthly irrigated and rainfed crop areas around the year 2000: A new high-resolution data set for agricultural and hydrological modeling. Global Biogeochemical Cycles. 2010;24(1):n/a-n/a. doi: 10.1029/2008GB003435.
